# Effect of D-Cycloserine on the Effect of Concentrated Exposure and Response Prevention in Difficult-to-Treat Obsessive-Compulsive Disorder: A Randomized Clinical Trial

**DOI:** 10.1001/jamanetworkopen.2020.13249

**Published:** 2020-08-13

**Authors:** Gerd Kvale, Bjarne Hansen, Kristen Hagen, Jonathan S. Abramowitz, Tore Børtveit, Michelle G. Craske, Martin E. Franklin, Svein Haseth, Joseph A. Himle, Sigurd Hystad, Unn Beate Kristensen, Gunvor Launes, Anders Lund, Stian Solem, Lars-Göran Öst

**Affiliations:** 1 Bergen Center for Brain Plasticity, Haukeland University Hospital, Bergen, Norway; 2 Department of Clinical Psychology, University of Bergen, Bergen, Norway; 3 Center for Crisis Psychology, University of Bergen, Bergen, Norway; 4 Department of Psychiatry, Molde Hospital, Molde, Norway; 5 Department of Psychology and Neuroscience, University of North Carolina, Chapel Hill; 6 Department of Psychology, University of California, Los Angeles; 7 Rogers Memorial Hospital, Oconomowoc, Wisconsin; 8 Department of Psychology, University of Pennsylvania, Philadelphia; 9 Nidaros Outpatient Psychiatric Unit, St. Olavs Hospital, Trondheim, Norway; 10 School of Social Work, Department of Psychiatry, University of Michigan, Ann Arbor; 11 Department for Psychosocial Science, University of Bergen, Bergen, Norway; 12 Gaustad Hospital, Oslo University Hospital, Oslo, Norway; 13 Solvang Outpatient Psychiatric Unit, Sørlandet Hospital, Kristiansand, Norway; 14 Division of Psychiatry, Haukeland University Hospital, Bergen, Norway; 15 Department of Clinical Medicine, Section for Psychiatry, Faculty of Medicine and Dentistry, University of Bergen, Bergen, Norway; 16 Department of Psychology, Norwegian University of Science and Technology, Trondheim, Norway; 17 Department of Psychology, Stockholm University, Stockholm, Sweden

## Abstract

**Importance:**

Evidence is lacking for viable treatment options for patients with difficult-to-treat obsessive-compulsive disorder (OCD). It has been suggested that D-cycloserine (DCS) could potentiate the effect of exposure and response prevention (ERP) treatment, but the hypothesis has not been tested among patients with difficult-to-treat OCD.

**Objective:**

To evaluate whether DCS potentiates the effect of concentrated ERP among patients with difficult-to-treat OCD.

**Design, Setting, and Participants:**

The study was a randomized placebo-controlled triple-masked study with a 12-month follow-up. Participants were adult outpatients with difficult-to-treat OCD. A total of 220 potential participants were referred, of whom 36 did not meet inclusion criteria and 21 declined to participate. Patients had either relapsed after (n = 100) or not responded to (n = 63) previous ERP treatment. A total of 9 specialized OCD teams within the public health care system in Norway participated, giving national coverage. An expert team of therapists from the coordinating site delivered treatment. Inclusion of patients started in January 2016 and ended in August 2017. Data analysis was conducted February to September 2019.

**Interventions:**

All patients received individual, concentrated ERP treatment delivered during 4 consecutive days in a group setting (the Bergen 4-day treatment format) combined with 100 mg DCS, 250 mg DCS, or placebo.

**Main outcomes and Measures:**

Change in symptoms of OCD and change in diagnostic status. Secondary outcomes measures included self-reported symptoms of OCD, anxiety, depression, and quality of life.

**Results:**

The total sample of 163 patients had a mean (SD) age of 34.5 (10.9) years, and most were women (117 [71.8%]). They had experienced OCD for a mean (SD) of 16.2 (10.2) years. A total of 65 patients (39.9%) were randomized to receive 100 mg DCS, 67 (41.1%) to 250 mg of DCS, and 31 (19.0%) to placebo. Overall, 91 (56.5%) achieved remission at posttreatment, while 70 (47.9%) did so at the 12-month follow-up. There was no significant difference in remission rates among groups. There was a significant reduction in symptoms at 12 months, and within-group effect sizes ranged from 3.01 (95% CI, 2.38-3.63) for the group receiving 250 mg DCS to 3.49 (95% CI, 2.78-4.18) for the group receiving 100 mg DCS (all *P* < .001). However, there was no significant effect of treatment group compared with placebo in obsessive-compulsive symptoms (250 mg group at posttreatment: *d* = 0.33; 95% CI, −0.10 to 0.76; 100 mg group at posttreatment: *d* = 0.36; 95% CI, −0.08 to 0.79), symptoms of depression and anxiety (eg, Patient Health Questionnaire–9 score among 250 mg group at 12-month follow-up: *d* = 0.30; 95% CI, −0.17 to 0.76; Generalized Anxiety Disorder–7 score among 100 mg group at 12-month follow-up: *d* = 0.27; 95% CI, −0.19 to 0.73), and well-being (250 mg group: *d* = 0.10; 95% CI, −0.42 to 0.63; 100 mg group: *d* = 0.34; 95% CI, −0.19 to 0.86). No serious adverse effects were reported.

**Conclusions and Relevance:**

In this study, DCS did not potentiate ERP treatment effect, but concentrated ERP treatment was associated with improvement.

**Trial Registration:**

ClinicalTrials.gov identifier: NCT02656342

## Key Points

**Question:**

Does D-cycloserine potentiate the effect of concentrated exposure and response prevention in difficult-to-treat obsessive-compulsive disorder?

**Findings:**

In this randomized clinical trial of 163 participants, D-cycloserine did not significantly affect treatment outcomes. Most patients responded to the concentrated exposure and response prevention treatment, and nearly 50% were recovered at 1-year follow-up.

**Meaning:**

In this study, concentrated exposure and response prevention treatment was effective for patients with difficult-to-treat obsessive-compulsive disorder, but adding D-cycloserine did not potentiate the treatment.

## Teaser

This randomized clinical trial evaluates whether D-cycloserine potentiates the effect of concentrated exposure and response prevention among patients with difficult-to-treat obsessive-compulsive disorder.

## Introduction

Obsessive-compulsive disorder (OCD) is a debilitating psychiatric disorder with a lifetime prevalence of 1% to 2%,^1,2^ often with onset in childhood.^3^ Exposure and response prevention (ERP) and antidepressants are effective treatments,^4,5^ but response is mixed, indicating a need to develop more effective strategies. Based on experimental animal studies demonstrating that D-cycloserine (DCS), a partial N-methyl-D-aspartate receptor agonist,^6,7^ facilitates extinction learning, a number of human clinical trials have assessed whether ERP is augmented by DCS. While some early publications were promising,^8,9^ more recent meta-analyses show only small augmentation of DCS vs placebo (effect size, 0.25)^10^ and others no significant difference,^11,12,13^ including no significant difference in several studies involving patients with OCD.^10,11,14,15,16^ A study by Andersson et al^17^ concluded that DCS did not augment the effects of cognitive behavioral therapy, but found that antidepressants may interact with DCS to block its facilitating effect on fear extinction.

The DCS studies included in meta-analyses are based on samples in which a treatment response would be expected for 62% to 68% of the sample.^4^ Potentiation by DCS may be more evident in patients who have a documented history of being difficult to treat, ie, those who have not responded to ERP or who have responded but then relapsed. These patients also constitute the group most in need of alternative treatment approaches. One study^18^ reported that DCS accelerated the rate of recovery in children with difficult-to-treat OCD, suggesting that DCS might also be helpful for adults with difficult-to-treat OCD.

The present study targets patients with OCD who have a documented history of nonresponse to or relapse following ERP treatment and investigates whether DCS potentiates the effect of concentrated ERP treatment immediately after treatment and at 12-month follow-up. We hypothesized that patients receiving DCS would demonstrate significantly greater reductions in OCD symptoms relative to participants in the placebo group.

## Methods

Norwegian health authorities have established 15 specialized adult OCD teams, yielding national coverage. Nine teams recruited participants for the study. Data collection, management, training of therapists, and organization of treatment were conducted from the Bergen site. Details regarding the clinical training procedure are described elsewhere.^19,20^ A total of 8 group leaders and 64 therapists participated. The study was approved by the regional committees for medical and health research ethics in Norway, and all participants provided written consent. The trial protocol is available in Supplement 1. The study followed the Consolidated Standards of Reporting Trials (CONSORT) guideline.

### Design and Participants

All patients received concentrated ERP treatment delivered during 4 consecutive days in groups of 3 to 6 patients with a 1:1 ratio between patients and therapists. The 2 middle days were used for exposure treatment (eFigure 1 in Supplement 2). Effectiveness studies in routine clinical care^19,20,21,22^ as well as a randomized clinical trial^23^ have demonstrated that 90% of patients with OCD respond to ERP treatment delivered in this format and that 70% are recovered at 4-year follow-up,^24^ based on the international consensus criteria.^25^

DCS was administered both days of exposure treatment. Given that research on optimal dosage has been inconclusive,^10,13^ 100 mg and 250 mg dosages were evaluated. Participants were stratified by use of antidepressants.^17^ Thus, the study used a triple-masked, 3-group, placebo-controlled design, in which patients within each stratum were randomized to 100 mg DCS, 250 mg DCS, or placebo in a 2:2:1 ratio for an intended sample of 160. Randomization in blocks of 5 was done using an online tool before the first patient was included in the study and concealed from all patients, therapists, and independent assessors. Due to the group treatment format, the actual sample size was 67 of 163 (41.1%) in the 250 mg group, 65 (39.9%) in the 100 mg group, and 31 (19.0%) in the placebo group. The trial was announced through media and on the websites of the Norwegian OCD association and the OCD teams. Inclusion lasted from January 2016 to August 2017.

#### Inclusion Criteria

We included patients who met *Diagnostic and Statistical Manual of Mental Disorders* (Fifth Edition) (*DSM*-*5*) criteria for OCD; were able to be treated as outpatients; were aged at least 18 years; were fluent in Norwegian; and had either responded to and relapsed following or not responded to prior ERP treatment, consisting of at least 6 sessions of ERP. Response to earlier ERP was defined by an at least 35% reduction and a posttreatment Yale-Brown Obsessive Compulsive Scale (Y-BOCS) score of 15 or lower; relapse was defined by an at least 35% increase in Y-BOCS score from posttreatment, a Y-BOCS score of 16 or more, and a Clinical Global Impression (CGI) improvement score of 6 (ie, “much worse”) or higher.^25^ Nonresponders were defined as those with a reduction in Y-BOCS scores from pretreatment to posttreatment of less than 35% and a Y-BOCS score of at least 16 after treatment. A minimum of 4 weeks since treatment ended was required.

#### Exclusion Criteria

Patients who had ongoing substance abuse and/or dependence; had bipolar disorder or psychosis; had active suicidal ideation or plans; had not receiving a stable dosage of antidepressants for at least 12 weeks or were not willing to receive a stable dosage during the 4 intervention days; were unwilling to refrain from anxiety-reducing substances during the 2 days of exposure; had an intellectual disability; and were living more than 1 hour by car or train from the treatment location were excluded. Exclusion criteria related to the DCS were pregnancy or breastfeeding, kidney impairment, hypersensitivity to DCS, porphyria, and epilepsy.

### Determination of Eligibility

Trained assessors evaluated eligibility in terms of diagnosis, prior Y-BOCS scores, and the other inclusion and exclusion criteria listed. Three senior investigators (G.K., B.H., J.A.H.) evaluated and decided on questionable cases. However, this happened in only 1 case.

### Adherence and Competence

All group sessions and therapist meetings were videotaped. Also, each group had a trained therapist who observed and evaluated whether the group was conducted in accordance with the protocol. No deviances from the protocol were reported for any of the groups. Two experts on concentrated ERP who had not participated in the given group independently scored all videotapes for adherence and competency using a 3-point scale. With 1 exception, both experts rated all groups as adherent and competent.

### Independent Assessors

All Y-BOCS and Structured Clinical Interview for *DSM*-*5* (SCID-5) were conducted by specially trained and independent assessors and were audiotaped. A second assessor rated 20% of the taped interviews. The obtained κ coefficient of diagnostic agreement was excellent (κ = 0.92). The same procedure was used for the Y-BOCS interviews, and the interrater reliability of total score was excellent (intraclass correlation coefficient, 0.94).

### DCS

Each patient received 1 capsule of DCS (100 mg or 250 mg) or placebo each of the 2 days of exposure. DCS and placebo were prepared in identical capsules by a research pharmacy. The participants received written information about the medication and a phone number for questions or to report adverse events. Some prior trials have indicated that DCS might potentiate negative experiences when exposure is brief and not followed by a reduction in anxiety.^26^ To minimize this risk, the first capsule was taken when the patient had gained experience with the procedure, ie, at lunchtime on day 2.

At posttreatment, assessors were asked to guess which dosage of medication the patients had received and also to indicate how certain they were of their guesses. The result showed no correspondence between the actual group and guesses (χ^2^_4_ = 2.62; *P* = .62). Certainty was rated from 0 to 10, and the mean (SD) score was 4.0 (3.0). Patients were asked the same questions and the results showed a mean (SD) certainty of 0.3 (0.9), and no correspondence between the actual group and their guesses (χ^2^_4_ = 2.45; *P* = .65).

### Diagnostic Procedure

All patients were screened for inclusion using Y-BOCS and Mini International Neuropsychiatric Interview (MINI).^27^ Patients receiving a preliminary OCD diagnosis after the MINI had a diagnostic interview using the SCID-5.^28^ The SCID-5 was carried out by a team of masked independent assessors who had undergone extensive training in the procedure.

### Primary Outcome Measures

Patients were assessed pretreatment, posttreatment, and at 3-month and 12-month follow-ups. The Y-BOCS^29^ was the primary outcome measure. In addition to mean scores on Y-BOCS, clinical improvement was evaluated using a modified version (not including the CGI) of the international consensus criteria, which defines response as a reduction of at least 35% of pretreatment Y-BOCS score and remission as the response criterion plus a posttreatment Y-BOCS score of 12 points or lower.^25^ Recovery was defined as in remission at 1-year follow-up. We also used the criteria by Jacobson and Truax^30^ for clinically significant change; the results were very similar to those obtained with the international consensus criteria (eTable in Supplement 2). Finally, we reported change in diagnostic status (*DSM-5*) as assessed by SCID-5 at 3-month and 12-month follow-ups.

### Secondary Outcome Measures

We used 6 self-report scales. They were the Dimensional Obsessive-Compulsive Scale Short Form,^31^ the Obsessive-Compulsive Inventory–Revised,^32^ Generalized Anxiety Disorder–7 (GAD-7),^33^ the Patient Health Questionnaire–9 (PHQ-9),^34^ the Client Satisfaction Scale–8,^35^ and the Warwick-Edinburgh Mental Well-being Scale (WEMWBS).^36^

### Statistical Analysis

A meta-analysis^37^ found a moderate effect size for ERP with DCS for heterogeneous samples of patients with OCD. Given the inclusion of only cognitive behavioral therapy nonresponders or patients who had relapsed after ERP, we anticipated a larger effect size compared with previous ERP studies. To have 80% power to detect a moderate effect size (*d* = 0.50) at an α of .05, a total of 160 patients needed to be included (64 in each of the DCS groups and 32 in the placebo group). We compared Y-BOCS scores between the placebo and DCS (100 mg and 250 mg) groups from pretreatment to posttreatment and 2 follow-ups using mixed-effects regression models.^37^ The dropout at the different points of assessment was very low (Figure). Following the principle of intention to treat, all participants were included in the analyses, irrespective of missing data at any measurement point.^38^ Time was treated as a categorical variable because we did not expect a strictly linear effect of time. Between-group differences in Y-BOCS were assessed by including fixed effects for treatment group, time, and the time × treatment group interaction. Bonferroni confidence intervals were used for all analyses.

**Figure.**
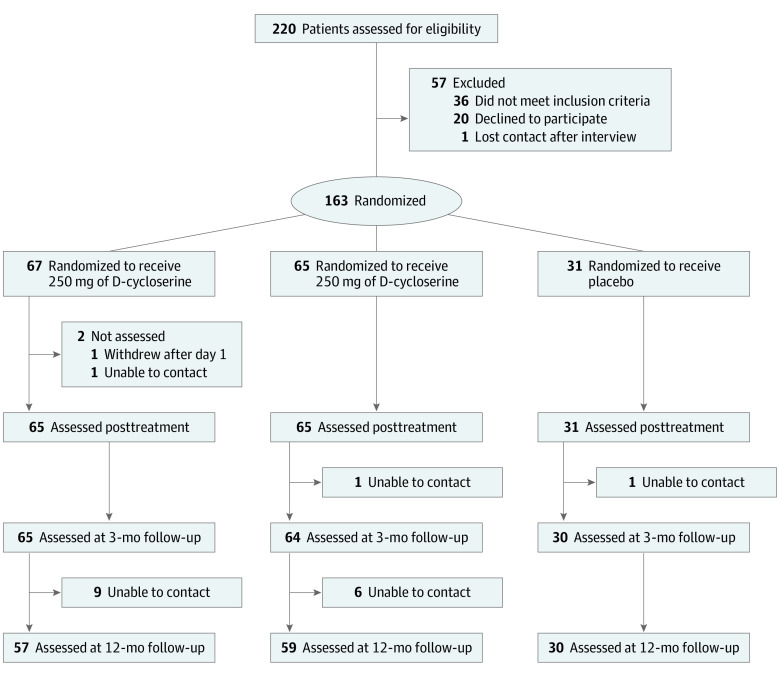
Study Flowchart

The mixed model included random intercepts for all participants. To take the potential clustering effect of group leader into account, we also included a random effect for group leader. Participants were also stratified according to use of antidepressants. To examine a possible effect of current use of antidepressants, a 3-way interaction among time, treatment condition, and current use of antidepressants was included in the analysis. Mixed models also allow the use of realistic variance and correlation patterns to achieve more efficient statistical inference and, therefore, greater statistical power.^38^ In this study, an unstructured covariance structure was used. This choice was informed by comparing plausible covariance structures using goodness-of-fit statistics (deviance, Akaike information criterion, and Bayesian information criterion).^38^ Intervention (within-group) effect sizes were estimated using Glass Δ, with pretreatment SD as denominator. The manipulation in intervention studies will often affect the SD as well as the mean; therefore Glass Δ is recommended.^39^ Effect size is commonly interpreted as small (0.2), moderate (0.5), and large (0.8).^39^

Data analysis was conducted from February to September 2019, using Stata version 16 (StataCorp) and SPSS statistical software version 25 (IBM Corp). Statistical significance was set at *P* < .05, and all tests were 2-tailed.

## Results

### Inclusion of Patients

The Figure presents the study flowchart. A total of 220 individuals were referred and assessed for eligibility. Overall, 57 (25.9%) were excluded for reasons shown in the Figure. Of the 163 included participants, 65 (39.9%) were randomized to receive 100 mg DCS, 67 (41.1%) to receive 250 mg DCS, and 31 (19.0%) to receive placebo.

### Pretreatment Characteristics

The total sample mean (SD) age was 34.5 (10.9) years, and most were women (117 [71.8%]). They had experienced OCD for a mean (SD) of 16.2 (10.2) years. They had moderate to severe symptoms of OCD and moderate symptoms of depression and generalized anxiety. A total of 100 (61.3%) had relapsed following their previous treatment for OCD, whereas 63 (38.7%) did not respond to their previous treatment. Overall, 72 (44.2%) received disability benefits, 56 (34.8%) were employed, and 33 (20.5%) were students. The total sample had attended school for a mean (SD) of 11.9 (3.9) years. A total of 64 participants (39.3%) reported having family members who also had OCD symptoms. Overall, 76 participants (46.6%) used psychotropic medication, of whom 52 (68.4%) used selective serotonin reuptake inhibitors. A summary of sample characteristics is shown in Table 1. There were no significant differences between the 3 groups except for mean (SD) previous pretreatment Y-BOCS scores; participants in the 250 mg group had a 2-point lower mean score than participants in the other 2 groups (DCS 250 mg group: 25.6 [4.8]; DCS 100 mg group: 27.7 [5.0]; placebo: 27.6 [5.1]).

**Table 1.** Pretreatment Characteristics of Participants by Treatment Group

Characteristic	Mean (SD)		
	250 mg (n = 67)	100 mg (n = 65)	Placebo (n = 31)
Age, y	34.82 (11.75)	35.38 (11.42)	32.42 (7.06)
Women, No. (%)	45 (67.2)	49 (75.4)	23 (74.2)
Years in school	11.75 (3.82)	11.44 (4.13)	12.90 (3.26)
Age of OCD onset, y	19.21 (10.29)	19.12 (10.70)	16.79 (7.22)
Duration of OCD, y	15.89 (9.56)	16.58 (10.87)	15.93 (10.26)
Previous treatment exposure, h	26.17 (11.55)	26.37 (10.54)	27.97 (10.73)
Previous pretreatment Y-BOCS score	25.61 (4.79)	27.69 (5.00)	27.61 (5.07)
Previous posttreatment Y-BOCS score	14.51 (6.63)	13.52 (5.48)	14.69 (5.98)
Current Y-BOCS score	26.66 (3.99)	27.26 (3.75)	27.35 (3.83)
Any comorbid disorder, No. (%)	46 (68.7)	47 (72.3)	20 (64.5)
Comorbid disorders, No.	1.91 (2.05)	1.71 (1.88)	1.48 (1.55)
DOCS-SF score	26.55 (6.35)	27.82 (5.04)	27.20 (6.45)
OCI-R score	28.42 (11.09)	28.74 (13.32)	28.33 (10.91)
PHQ-9 score	11.40 (6.21)	12.75 (6.18)	11.43 (4.51)
GAD-7 score	12.31 (4.72)	12.84 (4.01)	11.56 (4.65)
WEMWBS score	37.79 (8.69)	39.14 (8.12)	40.63 (6.71)
Outcome of previous treatment, No. (%)			
Nonresponder	29 (43.3)	23 (35.4)	11 (35.5)
Relapse	38 (56.7)	42 (64.6)	20 (64.5)
Self-reported OCD in the family, No. (%)	26 (38.8)	26 (40.0)	12 (38.7)
Employment, No. (%)			
Work	22 (33.3)	25 (38.5)	9 (30.0)
Student	18 (27.3)	8 (12.3)	7 (23.3)
Disability	26 (39.4)	32 (49.2)	14 (46.7)
Psychotropic medication, No. (%)	25 (37.3)	36 (55.4)	15 (48.4)
Antidepressants, No. (%)	22 (32.8)	22 (33.8)	8 (25.8)

Abbreviations: DOCS-SF, Dimensional Obsessive-Compulsive Scale–Short Form; GAD-7, Generalized Anxiety Disorder–7; OCD, obsessive-compulsive disorder; OCI-R, Obsessive-Compulsive Inventory–Revised; PHQ-9, Patient Health Questionnaire–9; WEMWBS, Warwick-Edinburgh Mental Well-being Scale; Y-BOCS, Yale-Brown Obsessive Compulsive Scale.

The mean (SD) score on WEMWBS (39.7 [8.1]) indicated reduced mental well-being among the participants (compared with normal population mean of 50). The mean (SD) score of 12.0 (5.9) on the PHQ-9 and 12.4 (4.4) on the GAD-7 also indicated that the participants experienced moderate symptoms of both depression and generalized anxiety. Most had comorbid diagnoses (113 [69.3%]), with the most common being generalized anxiety disorder (52 [31.9%]) and major depressive disorder (51 [31.3%]). The mean number of hours for the previous ERP treatment was 26.6 (SD, 11.0; range, 9-60).

### Number of Groups and Sites

There were a total of 36 groups with group sizes ranging from 3 to 6 patients. Nine clinics were involved in the trial, of which 4 (Bergen, Oslo, Kristiansand, and Trondheim) treated 125 patients (76.7%). There were no significant differences in Y-BOCS scores between the 4 clinics at the 4 points of assessment.

### Treatment Effect of DCS vs Placebo

Results on the primary and secondary outcome measures are presented in Table 2. To test the hypothesis that DCS (regardless of dosage) enhances ERP, we specified a contrast that compared the 2 DCS groups (250 mg and 100 mg) with the placebo group and interacted this with time. This interaction was not statistically significant (χ^2^_3_ = 6.23; *P* = .10). Treating the 2 DCS groups as independent also resulted in a statistically nonsignificant time × treatment group interaction (χ^2^_6_ = 7.59; *P* = .27). Effect sizes for the primary and secondary outcome measures are presented in Table 3.

**Table 2.** Primary and Secondary Outcome Measures

Outcome measure	Score, mean (SD)				*P* value for time × group
	Pretreatment	Posttreatment	3-mo Follow-up	12-mo Follow-up	
Y-BOCS					
250 mg DCS	26.66 (3.99)	12.09 (5.44)	13.03 (7.35)	14.63 (7.38)	.27
100 mg DCS	27.26 (3.75)	11.86 (5.44)	13.77 (6.97)	14.19 (7.09)	
Placebo	27.35 (3.83)	14.06 (7.13)	16.10 (7.23)	14.10 (7.57)	
DOCS-SF					
250 mg DCS	26.55 (6.35)	13.84 (7.28)	14.21 (7.97)	17.78 (8.55)	.61
100 mg DCS	27.82 (5.04)	14.40 (7.54)	16.05 (8.91)	18.49 (9.61)	
Placebo	27.20 (6.45)	16.27 (8.15)	18.25 (6.96)	19.38 (9.91)	
OCI-R					
250 mg DCS	28.42 (11.09)	10.26 (7.29)	11.70 (8.62)	18.14 (11.29)	.78
100 mg DCS	28.74 (13.32)	12.75 (8.67)	14.75 (10.56)	20.10 (13.29)	
Placebo	28.33 (10.91)	13.13 (11.65)	15.11 (9.77)	20.00 (12.86)	
GAD-7					
250 mg DCS	12.31 (4.72)	7.54 (5.83)	7.08 (4.93)	8.58 (5.09)	.006
100 mg DCS	12.84 (4.01)	7.97 (4.27)	8.32 (4.70)	9.48 (5.04)	
Placebo	11.56 (4.65)	7.97 (5.15)	10.29 (4.61)	10.92 (6.09)	
PHQ-9					
250 mg DCS	11.40 (6.21)	7.21 (6.01)	6.50 (5.44)	8.54 (5.88)	.45
100 mg DCS	12.75 (6.18)	8.82 (6.37)	8.95 (6.25)	10.36 (6.31)	
Placebo	11.43 (4.51)	7.70 (5.53)	8.54 (4.31)	10.31 (6.15)	
WEMWBS					
250 mg DCS	37.79 (8.69)	NA	NA	43.28 (8.82)	.97
100 mg DCS	39.14 (8.12)	NA	NA	41.12 (9.57)	
Placebo	40.63 (6.71)	NA	NA	44.15 (7.44)	

Abbreviations: DCS, D-cycloserine; DOCS-SF, Dimensional Obsessive-Compulsive Scale–Short Form; GAD-7, Generalized Anxiety Disorder–7; NA, not applicable; OCI-R, Obsessive-Compulsive Inventory–Revised; PHQ-9, Patient Health Questionnaire–9; WEMWBS, Warwick-Edinburgh Mental Well-being Scale; Y-BOCS, Yale-Brown Obsessive Compulsive Scale.

**Table 3.** Within-Group and Between-Group Effect Sizes at Posttreatment and 12-Month Follow-Up

Outcome measure	Within-group effect size^a^		Between-group effect size^b^	
	Posttreatment	12-mo Follow-up	Posttreatment	12-mo Follow-up
	Glass Δ (95% CI)	Glass Δ (95% CI)	*d* (95% CI)	*d* (95% CI)
Y-BOCS				
250 mg DCS	3.65 (2.93 to 4.35)	3.01 (2.38 to 3.63)	0.33 (–0.10 to 0.76)	–0.07 (–0.51 to 0.37)
100 mg DCS	4.11 (3.31 to 4.89)	3.49 (2.78 to 4.18)	0.36 (–0.08 to 0.79)	–0.01 (–0.45 to 0.42)
Placebo	3.47 (2.45 to 4.46)	3.46 (2.45 to 4.45)	NA	NA
DOCS-SF				
250 mg DCS	2.00 (1.51 to 2.48)	1.38 (0.95 to 1.80)	0.32 (–0.12 to 0.76)	0.18 (–0.28 to 0.64)
100 mg DCS	2.66 (2.08 to 3.23)	1.85 (1.37 to 2.32)	0.24 (–0.19 to 0.67)	0.09 (–0.37 to 0.55)
Placebo	1.69 (1.02 to 2.35)	1.21 (0.59 to 1.81)	NA	NA
OCI-R				
250 mg DCS	1.64 (1.18 to 2.09)	0.93 (0.54 to 1.31)	0.32 (–0.12 to 0.76)	0.19 (–0.30 to 0.62)
100 mg DCS	1.20 (0.79 to 1.60)	0.65 (0.28 to 1.01)	0.04 (–0.39 to 0.47)	–0.01 (–0.47 to 0.45)
Placebo	1.39 (0.77 to 2.00)	0.76 (0.20 to 1.32)	NA	NA
GAD-7				
250 mg DCS	1.01 (0.62 to 1.40)	0.79 (0.41 to 1.17)	0.08 (–0.36 to 0.51)	0.43 (–0.03 to 0.90)
100 mg DCS	1.21 (0.80 to 1.62)	0.84 (0.45 to 1.22)	0.00 (–0.43 to 0.43)	0.27 (–0.19 to 0.73)
Placebo	0.77 (0.20 to 1.33)	0.14 (–0.41 to 0.67)	NA	NA
PHQ-9				
250 mg DCS	0.67 (0.31 to 1.04)	0.46 (0.10 to 0.82)	0.08 (–0.35 to 0.52)	0.30 (–0.17 to 0.76)
100 mg DCS	0.64 (0.27 to 1.00)	0.39 (0.03 to 0.74)	–0.18 (–0.62 to 0.25)	–0.01 (–0.47 to 0.45)
Placebo	0.83 (0.27 to 1.37)	0.25 (–0.28 to 0.78)	NA	NA
WEMWBS				
250 mg DCS	NA	0.40 (–0.79 to –0.01)	NA	0.10 (–0.42 to 0.63)
100 mg DCS	NA	0.24 (–0.61 to 0.13)	NA	0.34 (–0.19 to 0.86)
Placebo	NA	0.52 (–1.10 to 0.06)	NA	NA

Abbreviations: DCS, D-cycloserine; DOCS-SF, Dimensional Obsessive-Compulsive Scale–Short Form; GAD-7, Generalized Anxiety Disorder–7; NA, not applicable; OCI-R, Obsessive-Compulsive Inventory–Revised; PHQ-9, Patient Health Questionnaire–9; WEMWBS, the Warwick-Edinburgh Mental Well-being Scale; Y-BOCS, Yale-Brown Obsessive Compulsive Scale.

^a^ Glass Δ was calculated by subtracting the mean posttreatment (or follow-up) score from the pretreatment score and dividing the result with the pretreatment SD.

^b^ Effect sizes (Cohen *d*) for between-group differences were calculated using the placebo group as a comparison group.

There was a significant main effect of time (χ^2^_3_ = 776.26; *P* < .001). The reduction in Y-BOCS score from pretreatment to posttreatment was significant for all treatment groups (placebo, –13.29 [95% CI, –16.53 to –10.05]; 100 mg DCS, –15.40 [95% CI, –17.63 to –13.17]; 250 mg DCS, –14.57 [95% CI, –16.80 to –12.34]). A statistically significant decrease in Y-BOCS at posttreatment was upheld at both follow-up assessments for all treatment conditions (eFigure 2 in Supplement 2).

Specific comparisons at the primary end points (posttreatment and 12-month follow-up) revealed no statistically significant differences on Y-BOCS score between the placebo group and the 2 DCS groups combined (posttreatment, χ^2^_1_ = 3.21; *P* = .07; 12-month follow-up, χ^2^_1_ = 0.07; *P* = .79). Patients with a history of nonresponse increased their Y-BOCS scores from posttreatment to the follow-up period (eFigure 3 in Supplement 2). The increase in Y-BOCS score from posttreatment to 3-month follow-up was statistically significant (2.43; 95% CI, 1.02 to 3.84), whereas the increase from 3-month to 12-month follow-up was not (1.56; 95% CI, –0.12 to 3.26). Nonresponders also had significantly higher Y-BOCS scores than those who had relapsed at posttreatment (2.34; 95% CI, 0.51 to 4.18), 3-month follow-up (3.89; 95% CI, 1.70 to 6.09), and 12-month follow-up (5.50; 95% CI, 3.26 to 7.73). To examine the effect of current use of antidepressants, a 3-way interaction between time, treatment group, and current use of antidepressants was included in the analysis. A joint test of the time × group × antidepressant interaction revealed no statistically significant interaction (χ^2^_6_ = 1.22; *P* = .98).

There were no significant differences among conditions when using the international consensus criteria at posttreatment (χ^2^_4_ = 3.35; *P* = .50) or 1-year follow-up (χ^2^_4_ = 4.73; *P* = .32), and there was no significant difference with respect to diagnostic status among groups at the 3-month follow-up (χ^2^_2_ = 1.38; *P* = .50) or 12-month follow-up (χ^2^_2_ = 0.24; *P* = .89) (Table 4).

**Table 4.** Clinical Improvement According to the International Consensus Criteria and Change in Diagnostic Status

Outcome	Group, No. (%)			
	DCS 250 mg	DCS 100 mg	Placebo	Total
**Clinical improvement**				
Posttreatment				
Remission	39 (60.0)	35 (53.8)	17 (54.8)	91 (56.5)
Response	15 (23.1)	22 (33.8)	7 (22.6)	44 (27.3)
No change	11 (16.9)	8 (12.4)	7 (22.6)	26 (16.2)
Total No.	65	65	31	161
12-mo Follow-up				
Remission	31 (50.8)	27 (47.4)	12 (42.9)	70 (47.9)
Response	10 (16.4)	7 (12.3)	5 (17.9)	22 (15.1)
No change	20 (32.8)	23 (40.3)	11 (39.2)	54 (37.0)
Total No.	61	57	28	146
**Diagnostic status**				
3-mo Follow-up				
OCD	27 (41.5)	27 (41.5)	16 (53.3)	90 (56.3)
No OCD	38 (58.5)	38 (58.5)	14 (46.7)	70 (43.8)
Total No.	65	65	30	160
12-mo Follow-up				
OCD	27 (47.4)	27 (45.8)	13 (41.9)	67 (45.6)
No OCD	30 (52.6)	32 (54.2)	18 (58.1)	80 (54.4)
Total No.	57	59	31	147

Abbreviations: DCS, D-cycloserine; OCD, obsessive-compulsive disorder.

There was a significant reduction in symptoms at 12 months, and within-group effect sizes ranged from 3.01 (95% CI, 2.38-3.63) for the group receiving 250 mg DCS to 3.49 (95% CI, 2.78-4.18) for the group receiving 100 mg DCS (all *P* < .001). However, there was no significant effect of treatment group compared with placebo in obsessive-compulsive symptoms at posttreatment (250 mg group: *d* = 0.33; 95% CI, −0.10 to 0.76; 100 mg group: *d* = 0.36; 95% CI, −0.08 to 0.79) and symptoms of depression and anxiety at the 12-month follow-up (eg, PHQ-9 score among 250 mg group: *d* = 0.30; 95% CI, −0.17 to 0.76; GAD-7 score among 100 mg group: *d* = 0.27; 95% CI, −0.19 to 0.73) (Table 3). There were also signs of improvement in well-being at the 12-month follow-up (250 mg group: *d* = 0.10; 95% CI, −0.42 to 0.63; 100 mg group: *d* = 0.34; 95% CI, −0.19 to 0.86). For all outcome measures, there were no significant time × group effects, except for generalized anxiety. The reduction in GAD-7 from pretreatment to posttreatment was significant for all treatment groups (placebo, –3.69 [95% CI, –5.99 to –1.38]; 100 mg DCS, –4.86 [95% CI, –6.39 to –3.32]; 250 mg DCS, –4.78 [95% CI, –6.32 to –3.23]). Unlike the 2 DCS groups, the GAD-7 score in the placebo group significantly increased from posttreatment to the 3-month follow-up (2.30; 95% CI, 0.14 to 4.47). At the 3-month follow-up, the mean GAD-7 score for the placebo group was also significantly higher than that of the 250 mg DCS group (mean difference, 3.11; 95% CI, 0.53 to 5.67) but not that of the 100 mg DCS group. There were no statistically significant differences between the groups at the 12-month follow-up.

### Client Satisfaction and Adverse Events

Participants were generally satisfied with the treatment as indicated by a mean score of 28.9 (SD, 3.63; median, 30; range 11-32) on the Client Satisfaction Questionnaire–8^35^ (maximum score is 32). A total of 25 patients reported 28 adverse events, as follows: headaches (9 [36.0%]), diarrhea (5 [20.0%]), constipation (1 [4.0%]), tiredness (2 [8.0%]), dizziness (1 [4.0%]), vomiting (2 [8.0%]), and pain (1 [4.0%]). The adverse effects were not systematically related to whether the patients had received DCS or placebo (9 patients [36.0%] from the 250 mg group, 10 [40.0%] from the 100 mg group, and 6 [24.0%] from the placebo group). No serious adverse effects were reported.

## Discussion

To our knowledge, this project represents the largest study testing a possible potentiation effect of DCS on ERP treatment for patients with OCD. While most previous DCS studies have included samples in which most participants can expect a clinical response to ERP treatment alone, the target group in the current study was patients with difficult-to-treat OCD, defined as patients who recently had received ERP and either not responded or responded and then relapsed. Thus, if DCS has a potentiating effect on ERP treatment, it could be expected to be detected in this group of patients. Also, this group of patients represented those who would be the most in need of a potentiation of the psychological treatment. The results showed that there was no indication that DCS potentiated the treatment response, neither at posttreatment nor at 1-year follow-up. Also, opposed to the study by Anderson et al,^17^ we did not find any significant effect of ongoing treatment with antidepressants on the effect of DCS.

### Limitations

This study has limitations. The lack of a DCS effect in the current study was not due to an overall inferior effect of the ERP treatment. Compared with means across 5 previous randomized clinical trials^8,9,14,16,24^ on ERP and DCS in patients with OCD, the present sample of patients with difficult-to-treat OCD started treatment with somewhat higher Y-BOCS scores (27.0 vs 25.9) but ended treatment at a very similar mean (12.5 vs 12.3). Furthermore, the posttreatment response rate of 135 patients (83.9%; ie, 91 patients who achieve remission and 44 patients with at treatment response) and remission rate of 56.5% (91 patients) compares favorably with rates reported in meta-analyses^4^ (response rate of 65.0% and remission rate of 50.0% for 15 samples).

In contrast to previous studies, the maskedness of the assessors was tested in the current study; their guesses of which treatment each patient had received was random. It is also noteworthy that only 1 patient dropped out of therapy and only 1 other did not complete the posttreatment assessment. Thus, selective attrition was not a problem in this study. Furthermore, the sample primarily consisted of women. Also, there was a slight difference between groups in pretreatment Y-BOCS scores.

The nonsignificant findings correspond with previous findings.^10,11,14,15,16^ The study was powered to detect an effect size of 0.50 because a smaller effect size would have limited clinical significance. This is especially the case given that the targeted group was patients with difficult-to-treat OCD. A 2019 meta-analysis^40^ suggested that more DCS doses (up to 9) and administering DCS more than 60 minutes before exposure were associated with better outcomes. However, the concentrated format used in the present study is not compatible with administering more than 2 doses. Previous research has also indicated that a possible effect occurs during treatment or immediately after treatment.^8,9,18^ Given our concentrated treatment format and having the first follow-up visit after 3 months, the theory of accelerated improvement was not tested. Given the nonsignificant results, we suggest that future research should test the suggestions brought forth by Rosenfield et al.^40^

## Conclusions

In this randomized clinical trial, DCS did not potentiate the effect of concentrated ERP for patients with difficult-to-treat OCD. However, concentrated ERP treatment was associated with improvement in symptoms of OCD, anxiety, and depression.

## Notes

Supplement 1. **Trial Protocol**

Supplement 2. **eFigure 1.** Outline of the 4-Day Treatment **eFigure 2.** Change Trajectory by Condition for the Yale-Brown Obsessive-Compulsive Scale **eFigure 3.** Change Trajectory by Relapse and Nonresponse for the Yale-Brown Obsessive-Compulsive Scale **eTable.** Clinical Improvement According to the Jacobson and Truax Criteria at Posttreatment and Follow-up

Supplement 3. **Data Sharing Statement**
