# Supplementary material for: Effect of D-Cycloserine on the Effect of Concentrated Exposure and Response Prevention in Difficult-to-Treat Obsessive-Compulsive Disorder: A Randomized Clinical Trial
Source: JAMA Netw Open. 2020 Aug 13;3(8):e2013249. doi: 10.1001/jamanetworkopen.2020.13249 (PMC7426745; doi:10.1001/jamanetworkopen.2020.13249)
Supplement: Supplement 1. — Trial Protocol [file jamanetwopen-3-e2013249-s001.pdf]

# Translational approach to the understanding and treatment of Obsessive-Compulsive Disorder (OCD). Can D-Cycloserine enhance and stabilize the treatment-response in relapsed and non-responding OCD-patients?
# A randomized, double-blind, placebo- controlled national study

| | |
|---|---|
| Product: | D-Cycloserine (250 mg and 100 mg |
| EudraCT Number: | 2013-002574-49 |
| Sponsors: | 1. Norwegian Research Council and Norway's Regional Health Authorities (project no.: 912013)<br>2. Helse Vest (project no: 911880) |
| Principal Investigator: | Gerd Kvale |

**Investigator agreement**:

I will provide copies of the protocol, any subsequent amendments and access to all information furnished by the sponsor to study personnel under my supervision .I will discuss this material with them to ensure that they are fully informed about the investigational study agent and the study protocol. I agree to conduct this clinical trial according to the protocol described herein, except when mutually agreed to in writing with the sponsor. I also agree to conduct this study in compliance with Good Clinical Practice (GCP) standards as defined by International Conference on Harmonization (ICH) Guidelines for Good Clinical Practice, all applicable national and local regulations, as well as the requirements of the appropriate Review Board/Independent Ethics Committee and any other institutional requirements.

**Investigator**

Signature Date

Name

Institution

Address

## Contents

**PROTOCOL SUMMARY** ..... **5**

**1. ABBREVIATIONS** ..... **7**

**2. ADMINISTRATIVE INFORMATION** ..... **9**

**3. BACKGROUND** ..... **11**

**4. DESIGN** ..... **16**

**5. INCLUSION AND EXCLUSION CRITERIA, WITHDRAWAL** ..... **17**

5.1 Inclusion Criteria ..... 17
5.2 Exclusion Criteria ..... 17
5.3 Withdrawal ..... 18
5.4 Subject Log ..... 18
6.1 Primary Endpoints ..... 19
*6.1.1* ***Changes in Y-BOCS*** ..... *19*
*6.1.2* ***Changes in diagnostic status*** ..... *19*
6.2 Secondary Endpoints ..... 19
6.3 Predictors ..... 19
6.4 Mediators ..... 20
6.5 Descriptives ..... 20
7. Description of the measurements employed ..... 20
7.1 Assessor administrated measurements. ..... 20
7.2 Self-report questionnaires ..... 21
*7.2.1* ***Self-report of symptoms and disorders*** ..... *21*
*7.2.2* ***Treatment characteristics*** ..... *22*
*7.2.3* ***Personal characteristics*** ..... *22*
7.3 Behavioral test ..... 23
7.4 Neuroimaging ..... 23

**8. PROCEDURAL CONSISTENCY** ..... **24**

8.1 Project organization ..... 24
8.2 Recruitment and attrition ..... 24
8.3 Self-report measures ..... 24
8.4 Therapist conducted screening and assessment ..... 24
8.5 Therapist competence and compliance ..... 25
8.6 Intervention: The concentrated ERP-treatment combined with DCS-administration ..... 25
9.1 Investigator meeting ..... 26
9.2 Training course in Good Clinical Practice ..... 26
9.3 Recruitment of participants ..... 26
9.4 Inclusion of participants ..... 26
9.5 Preparation for treatment ..... 26
9.6 Treatment ..... 26
9.7 Homework assignments during the first three weeks post treatment ..... 27
9.8 Follow-up visit to the clinic ..... 27
9.9 Post-treatment assessments ..... 27
10. DCS/ placebo side effects ..... 27
11. Measures to minimize bias ..... 28
11.1 Packaging, Labeling, Storage and Handling of Investigational Medicinal Products ..... 28

11.2 TREATMENT ASSIGNMENT/ RANDOMIZATION ..... 28
11.3 BLINDING AND CODE BREAKING ..... 29
11.4 CONCOMITANT AND PRIOR MEDICATION ..... 29
11.5 COMPLIANCE TO DRUG TREATMENT ..... 29
11.6 PRODUCT ACCOUNTABILITY ..... 29
11.7 CONTINUATION OF CET-TREATMENT IN DISCONTINUATION OF DCS-TREATMENT ..... 29
11.8 UNSCHEDULED VISITS TO THE CLINIC ..... 29
11.9 DISCONTINUATION OF THE STUDY ..... 30
11.10 CLINICAL SAFETY ASSESSMENTS ..... 30
11.11 LABORATORY ASSESSMENTS ..... 30

**12. PROCEEDINGS FOR ADVERSE EVENTS ..... 30**
12.1 DEFINITION OF ADVERSE EVENTS ..... 30
12.2 DEFINITION OF ADVERSE REACTIONS ..... 30
12.3 DEFINITION OF SERIOUS ADVERSE EVENTS ..... 30
12.4 DEFINITION OF SUSPECTED UNEXPECTED SERIOUS ADVERSE REACTIONS ..... 30
12.5 ASSESSMENT OF ADVERSE EVENTS ..... 31
*12.5.1 Assessment of Intensity ..... 31*
*12.5.2 Assessment of Causality ..... 31*
12.6 METHODS FOR ELICITING ADVERSE EVENTS ..... 31
12.7 REPORTING OF ADVERSE EVENTS ..... 31
*12.7.1 Reporting of Adverse Events ..... 31*
*12.7.2 Reporting of Serious Adverse Events ..... 31*
*12.7.3 Reporting of Suspected Unexpected Serious Adverse reactions ..... 31*
12.8 FOLLOW-UP OF ADVERSE EVENTS ..... 31

**13. STATISTICS AND DATA MANAGEMENT ..... 32**
13.1 STATISTICAL ANALYSIS ..... 32
13.2 DETERMINATION OF SAMPLE SIZE ..... 32

**14. QUALITY CONTROL AND QUALITY ASSURANCE ..... 32**
14.1 SOURCE DATA ..... 32
14.2 MONITORING ..... 32
14.3 PROTOCOL AMENDMENT ..... 33
14.4 INSPECTION ..... 33

**15. ETHICS ..... 33**
15.1 INDEPENDENT ETHICS COMMITTEE ..... 33
15.2 ETHICAL CONDUCT OF THE TRIAL (RISKS AND BENEFITS) ..... 33
15.3 SUBJECT INFORMATION AND INFORMED CONSENT ..... 33

**16. DATA HANDLING AND RECORD KEEPING ..... 33**
16.1 CASE REPORT FORMS ..... 33
16.2 RECORD KEEPING ..... 34

**17. FINANCING AND INSURANCE ..... 34**

**18. PUBLICATION POLICY ..... 35**

**REFERENCES: ..... 36**

**APPENDIX ..... 43**
TABLE 1. MEASUREMENTS EMPLOYED IN THE STUDY ..... 43
D-CYCLOSERINE, SUBSTANCE INFORMATION ..... 44

## Protocol Summary

| PROTOCOL IDENTITY AND OBJECTIVES | |
|---|---|
| EudraCT Number: | 2013-002574-49 |
| Protocol Title: | Translational approach to the understanding and treatment of Obsessive-Compulsive Disorder (OCD). Can D-Cycloserine enhance and stabilize the treatment-response in relapsed and non-responding OCD-patients?<br>A randomized, double-blind, placebo- controlled national study |
| Trial Objective: | Investigate whether DCS gives incremental effects to concentrated exposure in vivo with response prevention (ERP) in terms of reduced OCD symptoms and reduced relapse rate on follow-up in previously relapsed or non-responding OCD patients. |
| | |
| **INVESTIGATIONAL MEDICINAL PRODUCTS (1)** | |
| Test Product: | D-Cycloserine |
| Pharmaceutical Form: | 3-Isoxazolidinone, 4-amino-, (R)- (Formula: C3H6N2O2) |
| Route of Administration: | Orally |
| | |
| **METHODOLOGY** | |
| Trial Design: | A randomized, double-blinded placebo-controlled national study in difficult to treat OCD-patients. |
| Dose/Duration: | 1 capsule of 250 mg DCS or 100mg DCS or placebo on each of the two days with concentrated, individually tailored and therapist assisted cognitive behavior therapy (ERP). |
| Primary Endpoint: | Changes in OCD symptoms from pre-treatment (T0) to post-treatment (T1) as well as 3-months (T2), 12 months (T3) and 5-year follow-up (T4). |
| Efficacy Parameters: | Remission in diagnostic status according to the Structured Clinical Interview for DSM-5 Axis I disorders (SCID-I) criteria for OCD and<br>Clinician administered Y-BOCS interview by specially trained and blinded assessors. |
| Safety Parameters: | Adverse Events (AE) and Serious Adverse Events (SAE) assessed during the active treatment days, at post-treatment, 3-months (at clinic), 12 months and 5 years follow-up using telephone clinician assessments. |
| | |
| **POPULATION OF TRIAL SUBJECTS** | |
| Description of Trial Subjects: | Patients with a primary diagnosis of OCD according to the DSM-5 who have partially or fully relapsed, or not responded from standard ERP-treatment delivered by trained therapists. |
| Number of Subjects: | 160 patients with OCD |
| | |

| **TRIAL TIMETABLE** | |
|---|---|
| First Subject In: | October 2015 |
| Last Subject In: | June 2018 |
| | |
| 5 years follow-up | October 2020 - June 2023 |
| Last Subject Out: | June 2023. |

## 1. Abbreviations

| Abbreviation | Explanation |
|---|---|
| ADR | Adverse Drug Reaction |
| AE | Adverse Event |
| ANOVA | Analysis of variance |
| AR | Adverse Reaction |
| BIPQ | Brief Illness Perception Questionnaire |
| BIS | Bergen Insomnia Scale |
| BRIEF-A | Behavior Rating Inventory of Executive Function – Adult Version |
| cET | Concentrated Exposure-Based Treatment |
| CGI-I | Clinical Global Impression |
| CORE | Centre For Outcomes Research And Effectiveness |
| CRF | Case Report Form |
| CSRS | Clinical Severity Rating Scale |
| CSSRI | Client Socio-demographic and Service Receipt Inventory |
| DCS | D-Cycloserine |
| DRS-15-R | Dispositional Resilience Scale |
| DSM-IV | Diagnostic and Statistical Manual of Mental Disorders 4$^{th}$ edition |
| DSM-5 | Diagnostic and Statistical Manual of Mental Disorders 5$^{th}$ edition |
| DTAP | Difficult to treat anxiety patients |
| ERP | Exposure with Ritual Prevention |
| GAD-7 | Generalised Anxiety Disorder Assessment 7 |
| GAF | Global Assessment of Functioning |
| GCP | Good Clinical Practice |
| HSPS | Highly Sensitive Person Scale |
| IMP | Investigational Medicinal Products |
| M | Mean |
| M.I.N.I. | The Mini-International Neuropsychiatric Interview |
| MPA | Medicinal Product Agency |
| NML-2 | Nijmegen Motivational List-2 |
| NOMA | Norwegian Medicines Agency |
| NMDA | N-Methyl-D-Asparte |
| OCD | Obsessive Compulsive Disorder |
| OCI-R | Obsessive compulsive inventory – revised |
| PEAS | Patient Exposure and Response Prevention Adherence Scale |

| | |
|---|---|
| PHQ-9 | Patient Health Questionnaire 9 |
| PID-5 BF | Personality Inventory for DSM-5 – Brief Form |
| PTSD | Post-Traumatic Stress Disorder |
| RCI | Reliable Change Index |
| REC | Regional Committee for medical and Health Research Ethics |
| SADR | Serious Adverse Drug Reaction |
| SAE | Serious Adverse Event |
| SCID-I | Structured Clinical Interview for DSM-5 Axis 1 disorders |
| SD | Standard Deviation |
| SPC | Summary of Product Characteristics |
| SUD | Subjective Unit of Discomfort |
| SUSAR | Suspected Unexpected Serious Adverse Reaction |
| TC | Treatment Credibility |
| WAI | Working Alliance Inventory |
| WEMWBS | Warwick-Edinburgh Mental Well-being Scale |
| W & SAS | Work and Social Adjustment Scale |
| Y-BOCS | Yale-Brown Obsessive-Compulsive Scale |

## 2. Administrative Information

**Sponsors**: 1. Norwegian Research Council and Norway's Regional Health Authorities (project no: 912013) 2. Helse Vest (project number: 911880)

**Principal Investigator:**

**Gerd Kvale** (PhD, Professor), Bergen/ Haukeland University Hospital, Division of Mental Health, Tel: 0047- 91 63 86 81. E-mail: gerd.kvale@helse-bergen.no
Address: OCD-team, Haukeland University Hospital, Post Box 1400, N-5021 Bergen

**Co-Principal Investigator:**

**Bjarne Hansen** (PhD, Associate Professor). Tel: 0047- 92 09 07 65. E-mail: bjarne.kristian.aaslie.hansen@helse-bergen.no Address: OCD-team, Haukeland University Hospital, Post Box 1400, N-5021 Bergen

**Administrative project leader:**

**Kristin Odfjell** (Health Promoter), Tel: 0047- 99 16 84 09. E-mail: kristin.ostervold.odfjell@helse-bergen.no Address: OCD-team, Haukeland University Hospital, Post Box 1400, N-5021 Bergen

**Partners:**

| | |
|---|---|
| Oslo University Hospital: | Divisional manager Marit Bjartveit |
| Sykehuset Sørlandet: | Divisional manager Oddvar Sæter |
| Helse Møre og Romsdal: | Divisional manager Ketil Gaupset |
| St. Olavs Hospital: | Divisional manager Liv Sjøvold |

**Medical Competent:**

**Anders Lund** (Dr. Med., Professor), Bergen/ Haukeland University Hospital, Division of Mental Health, Tel: 0047- 55 95865600, E-mail: anders.lund@helse-bergen.no

**International expert team:**

**Professor Emeritus Michael Davis,** Emory University, Georgia
**Professor Jonathan Abramowitz,** University of North Carolina, Chapel Hill, NC
**Professor Emeritus Lars-Göran Öst,** Karolinska Institutet/ Stockholms Univeristy, Sweden
**Professor Michelle Craske,** University of California, Los Angeles, CA
**Professor Joseph A. Himle,** University of Ann Arbor, Michigan
**Associate Professor Marty Franklin,** University of Pennsylvania, Philadelphia, PA
**Associate Professor Odile van den Heuvel,** VU Medical Center, Netherlands

**Scientific Advisory Board:**

**Professor Ole Andreassen,** Oslo University Hospital/University of Oslo

**Professor Egil Martinsen,** Oslo University Hospital
**Professor Hans Nordahl,** Norwegian university of science and technology, NTNU, Trondheim
**Professor Ketil Ødegaard,** Haukeland University Hospital, Bergen
**Researcher Kitty Dahl,** Regional Centre for Child and Youth Mental Health and Child Welfare, region East/South.
**Head of Psychatric Ward/ PhD Vegard Haaland,** Department of Psychiatry, Sørlandet Hospital
**MD/ OCD-representative Erik Olsen,** Bergen
- **Arne Strand,** leader of OCD patient organization, Ananke

- **Web-page for the OCD-team:** www.helse-bergen.no/ocd

# 3. BACKGROUND

Approximately 30-40% of patients with anxiety disorders respond poorly to the documented effective exposure based psychological treatments and are not helped by traditional pharmacological interventions (4, 5). This group of Difficult to treat anxiety patients (DTAP) exceeds in number patients with psychosis or bi-polar affective disorders (6), and is overrepresented among the increase in the number of young disability cases seen in Norway the recent years (7, 8). The DTAP represent a clear challenge to the health care system and specialist health care in particular, where there are indications that 40% of the patients with treatment course of more than one year have anxiety as their main diagnosis, while the comparable figure for psychosis and bi-polar disorders are 15%. Improved help to the DTAP would have immense consequences for the individual patient as well as for the health care system and the society.

An obvious, but nearly unexplored possibility for the sub-optimal response to exposure-based treatments seen in the DTAP is that the variability in treatment response reflects fundamental differences in neural processing of emotional learning, and fear processing in particular. Fear-processing is dependent on N-methyl-D-aspartate (NMDA) receptors in amygdala and prefrontal cortex, and we argue that the combination of concentrated, prolonged exposure-based treatment and the NMDA agonist d-cycloserine (DCS) targeted to the DTAP might enhance and stabilize treatment effects for this challenging group of patients.

The current project thus combines basic neurobiological knowledge about fear relevant brain circuits with exposure-based psychological treatment, and a new generation of selectively targeted medications in order to develop a new treatment strategy for patients obviously not helped by our current approaches.

In order to reduce unwanted variability associated with diagnostic heterogeneity and at the same time target perhaps the most debilitating and complex anxiety disorder, we have chosen Obsessive Compulsive Disorder (OCD) as the model disorder. OCD is highly suitable for the suggested stringent experimental approach since the recommended treatment is analogous to what in basic laboratory research is labelled extinction. Furthermore, we have already developed a highly effective concentrated exposure-based treatment (cET) to be delivered during four consecutive days, which represent a unique experimental control over the treatment-condition. If the approach is successful, it will be extended to panic disorder, generalized anxiety disorder and Post Traumatic Stress Disorder (PTSD), as well as to children and adolescents.

**If our hypothesis is correct, the project might have direct implications treatment for patients with severe mental disorders.**

**Why a national project with OCD as the model disorder?**

Obsessive-Compulsive Disorder (OCD) is a chronic condition characterized by intrusive anxiety-provoking thoughts and images (obsessions), which the patients try to control by engaging in anxiety-reducing behaviors (compulsions). An example might be the fear of contamination, which the patient tries to regulate partly by avoiding all potential contaminants as well as by excessive washing rituals. The treatment of choice is called Exposure-in vivo with Response Prevention (ERP) which implies exposure to the anxiety provoking stimuli combined with full response prevention (no rituals) (2). Our recent meta-analysis (23) showed that 65% responded and 50% had a clinically significant change (remitted) from this approach.

At the moment there is a truly unique window which makes it possible to launch a national study on the DTAP, starting with OCD: The Norwegian Health authorities have decided that within a four-year period, starting in 2012, all patients with OCD shall have access to evidence-based psychological treatment through specialized OCD-teams (24). A large-scale training and supervision program, led by PhD/psychologist Bjarne Hansen together with internationally recognized OCD experts such as Jonathan Abramowitz, Martin Franklin and Joseph Himle, has been initiated. All the OCD-teams use the same protocol for screening, diagnostics, treatment, as well as for monitoring change and follow-up after treatment (24). This advantageous situation will place Norway, very soon, as the only country in the world with a high quality national database for OCD-patients covering diagnostics, treatment (procedure and process) outcome and follow-up data. These data can give valuable information about prior diagnosis and treatment outcome of eligible patients to be included in the study. Since all OCD-teams in this national implementation project basically use the same protocol for screening, diagnostics and treatment, as well standardized instruments for monitoring change during and after the interventions, the project represents a unique possibility for studying patients who have relapsed after initially successful treatment, or who has not obtained the expected effect.

The application is funded by the Norwegian Research Council with the following evaluation: "The project's objectives, research questions and hypotheses are very clearly presented and are based on an excellently formulated and highly original project concept. The project is in the forefront of its field and will contribute to scientific innovation as well as generate important new knowledge. The project is of excellent quality, with no significant weak points. It is a project at a very high international level and of great national and international interest".

**Basic emotional learning**

Fear is one of our most basic and important emotions: In order to survive, we have to rapidly learn what represents a danger (25, 26). Like Pavlov's dogs, which salivated to a sound previously paired with food, animals quickly can associate stimuli in their environment with actual or perceived life-threatening events (27). This basic emotional learning, often called "Pavlovian fear conditioning", is rapid and very long lasting, such as seen after extreme combat in humans, where fearful memories can last a lifetime. The neural circuit from the senses to amygdala and to behavior has been known for a long time, and the anatomy of fear learning is described by neuroscientists in great detail (26, 28).

When a conditioned bodily alarm is elicited, it creates a strong urge to escape or to avoid the situation – which is the core function of the fear learning (29). Crucial activities like eating, sleeping and having sex are terminated in the presence of fear, and as much as it is important to rapidly learn to avoid harmful situations, it is essential to be able to appropriately regulate the amount of fear to the degree of danger (30).

The defining feature of any anxiety disorder is the presence of excessive anxiety and discomfort in, or in anticipation of, basically unharmful or safe situations coupled with avoidance of these situations. Typically the person is aware that the anxiety is disproportional to the real danger or threat, but this insight does neither prevent nor stop the reaction; much like the salivation to the word "lemon" is hard to prevent once established, even though one may know that it is not adaptive in a situation where food is absent.

Although the conditioned reaction is elicited by situational cues – or by the mere thought of them - and thus beyond direct voluntary control, it is possible to exert voluntary control over the behavioral response, e.g. choose whether to avoid, escape or to stay in the situation (29). In line

with this, the focus of evidence-based psychological treatment is to help the patient to break the vicious cycle of elicited anxiety reactions and avoidance behavior by systematically helping the patient to confront the anxiety-provoking stimuli and to stay in the avoided situations, so-called exposure based therapies (4).

Exposure-based psychotherapy in humans is very similar to extinction training in animals (31, 32). When Pavlov's dogs after conditioning were presented repeatedly with the sound not followed by food, they eventually stopped salivating (33). Similarly, when a conditioned fear stimulus is presented repeatedly and not followed by a dangerous event, it also decreases in intensity and eventually ceases to occur at all (34) (35). Extinction is thus an important behavioral phenomenon that allows the organism to adapt its behavior to a changing environment.

Behavioral observations indicate that extinction is a form of learning in its own right, rather than an "unlearning" or forgetting of previous learning (36). Thus, after extinction training conditioned fear memories can return over time, when the fear stimulus is presented in a different context from the place where extinction training took place, or with an intervening stressor. This re-emergence of the fear response indicates that fear was not lost through extinction, but rather that fear was actively suppressed through an additional learning process. Thus, extinction is considered to be a form of acquired inhibition that counteracts or suppresses fear responses that are no longer adaptive (37, 38).

**Individual variability in emotional learning**

Despite knowledge from animal research (39), from human conditioning studies (40), as well as from clinical research (41) that there is a naturally occurring variability in Pavlovian conditioning (42) and in emotional fear processing (43, 44), this knowledge has been surprisingly underused in the efforts to develop new treatment strategies for anxiety disorders (45). In the following we argue that this variability might be of crucial importance, particularly for patients who have failed to respond to the existent evidence-based approaches.

**The role of NMDA receptors in fear extinction**

Fear conditioning is dependent on the amygdala, a brain area in the temporal lobe that receives indirect highly processed sensory information from cortex and the hippocampus, as well as direct, fast but poorly processed sensory information direct from the thalamus (46). The efferent part of amygdala projects to numerous other subcortical brain areas involved in the various responses that prepare and remove the animal from danger.

The N-methyl-D-aspartate (NMDA) receptor is a synaptic protein that binds the neurotransmitter glutamate and has been implicated in learning and memory in a variety of situations. Fear extinction also depends on NMDA receptors in both the amygdala and prefrontal cortex. Basic animal research indicates that if these receptors are blocked in the amygdala, prior to extinction training, fear extinction fails to occur (47, 48). If they are blocked in the prefrontal cortex, right after extinction training, fear extinction is not retained (49). D-cycloserine (DCS) is an analogue of the neurotransmitter, D-serine.

Like serine or another amino acid, glycine, it binds to a site on the NMDA receptor and facilitates the ability of glutamate to open the NMDA channel. When DCS is infused into the amygdala or given systemically, in conjunction with extinction training, it facilitates the rate of fear extinction (47). When infused into the prefrontal cortex it facilitates the retention of extinction (50). These beneficial effects never occurred if DCS was given alone, in the absence

of extinction training. Thus, DCS is not an anxiolytic; it has no beneficial effect on its own. Instead it facilitates the learning that takes place during extinction training.

Based on animal studies it was predicted and shown that DCS would facilitate exposure therapy in humans with acrophobia ("fear of heights")(9). This has been replicated in all the major anxiety disorders, including OCD (11, 12, 51-55) but with varying degrees of success. In some OCD studies the effect has clearly been observed in the beginning of the treatment (51), whereas other studies involving DCS and exposure for OCD have failed to show enhanced DCS-effects altogether (12, 14). DCS has been employed together with exposure based treatments in dosages from 50-100 mg, and since there are no conclusive results for dose-response relationships, the current study will administer DCS in both in 100 mg and 250 mg dosages.

So far, no good explanations have been provided for the variability in DCS response seen in the clinical settings, and in the following section we argue that the clinical usefulness of DCS has yet has to be fully tested. Below we specify necessary methodological requirements for studying a DCS effect that is clinically relevant.

**From animal studies to the clinic: How to elucidate a relevant DCS-effect in a clinical setting?**
***Ensure that the DCS-effect is not "overshadowed".*** Since most patients with anxiety disorders respond adequately to exposure-based treatments, extra stimulation of the NMDA-receptors might not be needed in order to gain a clinically relevant and stable change. Thus, even if DCS in a laboratory setting "speeds up" the treatment response and improves its consolidation, these effects might not be large enough to be seen, or elucidated in a clinical setting where the effect of exposure-based treatments alone would be sufficient to ensure clinically relevant changes. Given individual variation in the basic fear- and safety learning, individuals with a less pronounced response to psychological treatments, might be the ones who could be expected to profit from the added effect of DCS. The studies so far published on the combination of DCS and exposure based treatments have not made a distinction between patients who might be expected to be responders to the psychological intervention alone, or not. This implies that a possible DCS-response most likely be overshadowed/hidden by the effect of the exposure-based treatments alone (13, 56, 57). We are encouraged in this argument based on a recent study showing that DCS in fact did facilitate exposure-based treatments in a pilot study with children and adolescents where a substantial amount had been through previous treatment courses (54).

***Reduce unwanted variability related to diagnostics by using a model disorder.*** OCD, chosen as a model disorder and starting point, is characterized by intrusive anxiety-provoking obsessions, which the patients try to escape by engaging in compulsions. The effective treatment implies that the patient systematically approaches the situations/ stimuli that trigger anxiety while refraining from anxiety-reducing rituals. Numerous controlled studies of this treatment approach have demonstrated that between 60-70% of the patients can be expected to have substantial clinical gain from this approach (3, 23). OCD is classified as one of the most debilitating anxiety disorders (58) and is suitable as a model disorder both due to its severity, its complexity and at the same simplicity since the patients typically present highly specific anxiety triggers combined with rituals and avoidance which makes the treatment analogous to basic extinction training.

***Ensure that extinction is initiated.*** Since DCS has no effect on extinction on its own, but speeds up and consolidates extinction once it has started, it is a prerequisite to use an effective exposure-based treatment that will reliably initiate within-session extinction. Based on knowledge from one-session treatment of specific phobia, we have developed a concentrated exposure-based treatment with duration of only four consecutive days of which the two middle days constitute the active exposure-based treatment (cET). The treatment is individually tailored and covers a

wide range of relevant anxiety-triggers in different settings. A recent pilot (16) as well as an open trial study (59) as well as a systematic replication (60) indicate that all patients who have participated in the treatment have had within session anxiety reduction. By now >150 patients have been treated with this approach, and so far none have failed to show inter-session anxiety-reduction , even though nearly 20% show partial or full relapse at six months follow up. It is important to note that also patients with a previous history of being non-responders to exposure-based treatment show an initial response to this concentrated format.

***Reduce unwanted between-session variability***. As opposed to a laboratory setting where between-sessions events are controlled for, in the natural environment, patients will face naturally occurring exposures and might choose to deal with them by initiating anxiety-reducing behavior between sessions, which could jeopardize treatment outcomes. Usually such potentially crucial variability is impossible to control, but our cET format, however, elegantly deals with this challenge. Also, since the intervention is delivered during four consecutive days, drop-out is basically non-existent.

## The objective of the current study

**Objective:** To experimentally test a new treatment approach for the DTAP treated in the Specialist health care

**How:** This will be achieved by combining the individually tailored cET in a group format (maximum six patients in each group, 1:1 patient: therapist ratio) with DCS/ placebo administrated to patients with Obsessive Compulsive Disorder who have failed to respond to or relapsed after receiving ERP treatment

**Hypothesis**: Both DCS (100 and 250 mg) and placebo groups will show a significant improvement, but the patients who receive DCS will maintain the treatment effect significantly better than the placebo group. Thus, differences will occur at the follow-ups.

## 4. Design

The current study is a randomized, double-blind factorial design with DCS (250 or 100 mg) or placebo as between factors and assessment points as repeated factor. All patients receive concentrated ERP treatment. Assessments will be performed at baseline (T0), post-treatment (T1), and at follow-up after 3 (T2) and 12 months (T3) as well as after 5 years (T4).

For overview of study design, see Figure 1.

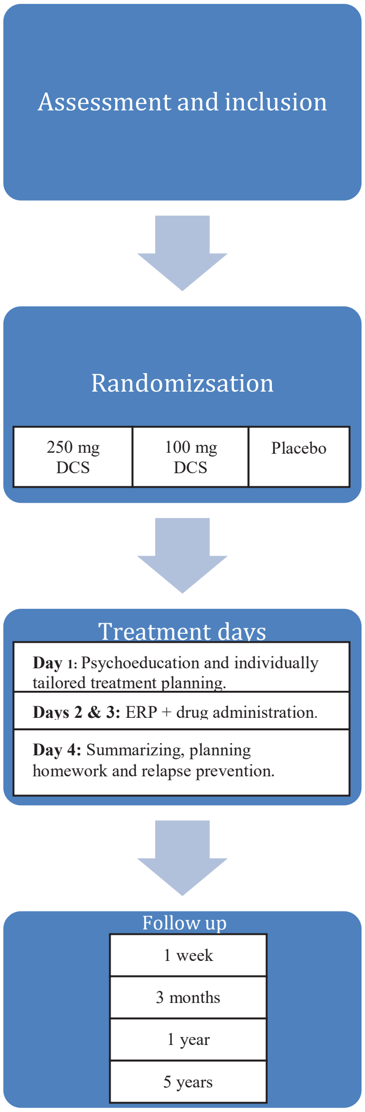


Figure 1 *Overview of the study design*

## 5. Inclusion and exclusion criteria, withdrawal

All 15 OCD-teams in Norway refer patients to the study, thus providing nationwide coverage of relapsed or non-responsive OCD patients.

A maximum of 160 patients will be included (see p.31 for power calculations) within the period from October 2015- June 2018.

Through the National OCD-implementation project all OCD-patients in Norway are ensured access to evidence based treatment. Standardized procedures for collection and storage of data (quality databases) are also established. These data will serve as reference data for standard treatment response, as well as provide detailed information regarding the first treatment-course for the relapsed OCD-patients.

The inclusion procedure implies that all relevant patients in Norway in the given time period will be offered participation. If this population turns out to be fewer than 160 patients during the study period, the inclusion period might be extended six months. If less than 160 patients are included at that point, analyses will be performed on the number of patients included by the end of the study period.

### 5.1 Inclusion Criteria

- Outpatients
- ≥ 18 years
- Fulfilling diagnostic criteria of OCD according to the DSM-5
- Previously have received ERP-treatment delivered by trained therapist and either have responded and relapsed, or not responded to the treatment.
    - Response is defined by ≥35% reduction with a post-treatment Y-BOCS score of 15, followed by a relapse as defined by > 35% increase in Y-BOCS score from post-treatment, a Y-BOCS score of 16 or more, and a CGI-I score of 6 ("much worse") or higher.
    - Non-responders are defined as those with a reduction in Y-BOCS scores from pre- to post-of less than 35%, and with a minimum Y-BOCS score of ≥16 after treatment. In order to be classified as non-responder as opposed to “drop-out” the patient has to previously have received a minimum of 6 sessions.
- There must be a minimum of 4 weeks since treatment ended.
- Fluent in Norwegian
- Signed informed consent

### 5.2 Exclusion Criteria

***Exclusion criteria related to the ERP***

- OCD symptoms primarily associated with hoarding
- Ongoing substance abuse/dependence
- Bipolar disorder or psychosis
- Ongoing/ planned suicidal ideation
- Mental Retardation, based on previous medical history
- If using antidepressants:
    - Not on stable dosage 12 weeks before the intervention
    - Unwilling to remain on stable dosage during the four intervention days

- Unwilling to refrain from anxiety reducing substances, such as anxiolytics (e.g. benzodiazepines) and alcohol during the two days of exposure.
- Patients living > 1 hour drive by car/ train from the treatment location.

***Additional exclusion criteria related to the DCS***

- Pregnancy or breast feeding (the participants are informed that they will have to use contraception the two days when the DCS/placebo is administered. Females will be asked if they are pregnant, and in case of doubt a pregnancy test is provided)
- Renal impairment
- Hypersensitivity to D-Cycloserine
- Porphyria
- Epilepsy

According to the SPC, depression and anxiety should also be exclusion criteria for the administration of DCS. However, in the current study DCS will be administrated in a low dosage for two consecutive days. Several studies have used DCS in the treatment of OCD with the same kind of exclusion criteria, and reported no adverse side effects [8, 9, 62], and studies of other anxiety conditions show the same results [6, 64].

### 5.3 Withdrawal

***Criteria for mandatory discontinuation of treatment, including DCS administration:***

- Consent withdrawal by the patient
- High suicide risk according to investigator's judgment or any suicide attempts during the two days administration of the DCS. These patients will be excluded from the study, and taken care of according to the normal procedures of specialist health care in Norway.
- Worsening of OCD symptoms during the two days of DCS-administration, which in the opinion of the investigator requires an adaptation of treatment not compatible with the protocol or requires hospitalization.

***Other possible reasons for premature discontinuation of treatment***

- Any serious adverse event or circumstances during the two days DCS administration justifying the discontinuation of the treatment in the investigator's opinion, if the serious adverse event is considered to be uniquely caused by DCS/placebo.
- Protocol deviation which jeopardizes the patient's safety.
- Patient lost to follow-up: When the participant discontinues reporting to the investigator, he/she must make an effort to contact the participant to establish the reason for discontinuation, and to suggest the participant comes to an end-of-study visit. If all such attempts to contact the participant fails, the investigator then declare the participant "lost to post-treatment assessment" and the investigator should document all these attempts in the corresponding medical file.

### 5.4 Subject Log

If possible, the investigator must record the reason and the exact time of the premature discontinuation of treatment in the case report form (CRF). If more than one reason is given, the investigator must indicate the main reason.

In case of treatment discontinuation for any reason between visits T0 and T2, an end-of-study visit should be done with all the assessments planned for the follow up as early as possible after the treatment study discontinuation.

# 6. Outcomes, predictors and mediators

For description of the measurements employed, see section 7. For overview of the times of measurements, see Table 1 (appendix).

## 6.1 Primary Endpoints

### 6.1.1 Changes in Y-BOCS

Changes in Y-BOCS from baseline (T0) to one week post treatment (T1), 3 months (T2) and 12 months (T3) as well as 5 years follow-up will be measured by specially trained and blinded raters, and calculated by two approaches which will be compared:

1. *Changes as suggested by an international consensus panel (61): Response* is a ≥35% reduction of the individual patient's pre-treatment YBOCS score. A patient is *remitted* if the response criterion is fulfilled and the post-treatment Y-BOCS score is ≥12 points.

2. *The criteria of Jacobson and Truax* (62) for clinically significant improvement: First, the change from pre- to post-assessment must be large enough to be statistically reliable at the 5%-level (Reliable Change Index; RCI). Second, a cut-off score is used: the patient's post-treatment score must be within the distribution of the normal population defined as M+2SD, *or* outside the distribution of the patient population defined as M-2SD. A *non-responder* is defined as not fulfilling the RCI, a *partial responder* as fulfilling the RCI but not the cut-off score, and a *full responder* as fulfilling both the RCI and the cut-off score. Response will.

### 6.1.2 Changes in diagnostic status

Diagnostic status according to the DSM-5 criteria for OCD at baseline and 3, 12 months and 5 years follow-up will be employed. The Structured Clinical Interview (SCID) for DSM-5 Axis I disorders will be employed (63).

## 6.2 Secondary Endpoints

***Self-report measures***

Measures of anxiety, depression, global functioning, severity of the disorder, self-reported OCD-symptoms, SUD-measures, quality of life, changes in work and social status as well as changes in medication and use of health care will be included as secondary end-points related to the main objective of the study. For description of instruments, see section 7 and Table 1 in the Appendix.

***Behavioral test***

The exposures during treatment will follow a specific procedure training the patient to "lean into the anxiety" (LET-technique, see description of instruments). The first and last exposure during the two treatment days will be videotaped and changes in patients behavior as well as self-reported anxiety scores and "holding back"-scores will be compared.

## 6.3 Predictors

The following variables will be employed as predictors of outcome: the patients' evaluation of the credibility of the treatment (73), presence of comorbidity (74, 75), number of previous

treatments, treatment motivation as well as the onset, duration and severity of the disorder. For description of instruments, see section 7. For assessment points see Table 1 in the Appendix.

### 6.4 Mediators

The working alliance, score of LET intervention during sessions as evaluated by the patient (76), within session reduction of anxiety, treatment evaluation, as well as compliance with homework after the treatment will be employed as mediators. For description of instruments, see section 7. For assessment points see Table 1 in the Appendix.

### 6.5 Descriptives

Please note that patients in the current trial are entitled to treatment in the Norwegian specialist health care, and that the questionnaires listed below are part of standard diagnostic and assessment procedures in the OCD-team in Helse-Bergen, and are approved as part of the current trial by the Regional Committee of Ethics (2013/195/REK Sør-Øst- D). The following are routinely measured: sleep disturbances (77); the patients' beliefs about the stability of the condition (78); stable personality characteristics (79); sensitivity to sensory and external stimulation (80); and Behavior Rating Inventory of Executive Function – Adult Version (82). For description of instruments, see section 7. For assessment points see Table 1 in the Appendix.

## 7. Description of the measurements employed

### 7.1 Assessor administrated measurements.

- *The Mini-International Neuropsychiatric Interview* (M.I.N.I.) (83) is a brief structured diagnostic interview, administered by a clinician, covering Axis 1 diagnoses in DSM-IV and ICD-10. The M.I.N.I. has been reported to have good psychometric properties (84).

- *Inclusion/exclusion evaluation:* A study specific CRF summarizing all inclusion and exclusion criteria. The form is to be used as a checklist by the clinician.

- *Structural Clinical Interview for DSM Disorders* [SCID-I (75, 85, 86)] cover Axis I psychiatric disorders according to DSM-5.

- *Yale-Brown Obsessive-Compulsive Scale* (Y-BOCS) (87). The Y-BOCS is regarded as the gold standard for assessing the severity of OCD symptoms. It consists of a symptom checklist covering obsessions and compulsions and a severity scale (88). The severity scale comprises 10 items, rated on a 5-point Likert scale ranging from 0 (no symptoms) to 4 (severe symptoms). The total score ranges from 0 to 40 and consists of sub scores for obsessions (range 0 to 20) and compulsions (range 0 to 20). Y-BOCS has excellent inter-rater reliability and moderate to good internal consistency. The Y-BOCS consists of both a clinical interview and self-report scale (87).

- *Global Assessment of Functioning* (GAF) (89) is used to evaluate global improvement in both symptoms and functioning. The range on each scale is from 0-100, with higher scores indicating better functioning.

- *The Clinical Global Impressions Scale* (CGI-I) (90). The CGI is a 8-point scale that requires the clinician to rate the severity of the patient's change/relapse at the time of assessment relative to the subjects condition after last treatment (baseline), with following

alternatives 0=Not assessed, 1 =Very much improved, 2 =Much Improved, 3=Minimally Improved, 4=No change, 5=Minimally worse, 6=Much worse, 7 =Very much worse

- *Evaluation of the treatment* is recorded (the therapist interviews the group members together) the last treatment day on covering different aspects of treatment satisfaction (16).

- *Anamnestic information and interview regarding latest treatment course.* Consists of a short relapse interview on how the previous treatment as well as the time after treatment was experienced by the patient, as well as a timeline for relapse.

- *CORE* Problem-specific therapist competences describe the knowledge and skills needed when applying CBT principles for OCD. Developed by Steketee, Foa and Kozak.

## 7.2 Self-report questionnaires

### 7.2.1 Self-report of symptoms and disorders

- *Obsessive-Compulsive Inventory – Revised* (OCI-R) (91). The OCI-R is an 18 items self-rating scale, measuring six different symptom dimensions of OCD. The OCI-R total score has high test-retest reliability and high sensitivity to change, in relation to the Y-BOCS (92).

- *Dimensional Obsessive Compulsive Scale Short-Form (DOCS-SF)* is a self-report questionnaire adapted from DOCS (93). It consists of a symptom checklist covering obsessive and compulsive thoughts about “contamination”; “responsibility for harm, injury or bad luck”; “unacceptable obsessional thoughts”; “symmetry completeness and exactness”. For all dimensions five items on scale from 0 to 8 assesses severity: (a) time occupied by obsessions and compulsions, (b) avoidance behavior, (c) associated distress, (d) interference with daily functioning, and (e) difficulty disregarding obsessions and refraining from the compulsions. Item A and E is split into two questions. The total score ranges from 0 to 40.

- *Generalized Anxiety Disorder Assessment* 7 (GAD-7) (94-96) is a brief 7-item self-report measure for generalized anxiety symptoms. Although developed for measuring severity of generalized anxiety disorder, the GAD-7 measures severity of general anxiety symptoms common to several anxiety disorders. The GAD-7 has been demonstrated to have good psychometric properties.

- *Patient Health Questionnaire,* (PHQ-9) (98-101) is a 9-item self-report questionnaire measuring level of depressive symptoms. Items are scored on a scale from 0 (not at all) to 3 (nearly every day). The PHQ-9 has been validated for clinical use and shows sound psychometric properties with high test-retest reliability.

- *The Bergen Insomnia Scale* (BIS) (77) will be used to screen for sleep disturbances. The scale has six items covering different aspects of sleep disturbances the past 4 weeks. The scale has acceptable psychometric properties (77)

- *Record of concomitant drug/medication* use during the period of the study will be recorded on a specially designed sheet.

- *Nijmegen Motivational List 2* (NML-2) is a 25-item questionnaire assessing how the disorder impacts on the patients, motivation for treatment and beliefs about the present form of therapy. It has acceptable psychometric properties (105).

### 7.2.2 Treatment characteristics

- *The Treatment Credibility Scale* (TC) assesses how credible the participants perceive the treatment to be and how much they expect that they will improve from the treatment. The first four items are rated on a 0-10 point scale, and the last rated in percentage (0-100%), higher scores indicating higher credibility ratings.

- *Evaluation of treatment.* (See section 7.1; Assessor administrated measurements.)

- *The Working Alliance Inventory* (WAI-P) (97) is a 12-item questionnaire that assesses the therapeutic relationship. The WAI has good psychometric properties.

- *LET (Leaning in technique)* scoring sheet measures patient's cognitions and behavior during exposure, and whether they are actively trying to embrace the experienced anxiety or instead trying to hold back and down regulate it.

- *Client Satisfaction Questionnaire (CSQ-8)(107)* eight-item self-report form where patients report their level of satisfaction with the treatment they have undergone.

- *Patient Exposure and Response Prevention Adherence Scale (PEAS)* (108) is a three-item scale where patients and clinicians separately report the quantity and how exposure and response prevention was performed. It has good psychometric properties, and has been shown to predict the efficacy of ERP (109).

- *Prioritized exposure tasks* is a specially designed worksheet where the patient records what he or she finds the most anxiety provoking in order to individually tailor the treatment for each patient.

- *Daily recording of exposure tasks* is a specially designed worksheet which the patient fills out each day for three consecutive weeks, and the logs are to be returned on a weekly basis. Each day the patients reports on seven different questions about the obsessive-compulsive disorder, compliance to homework and self-guided treatment progress. (16).

### 7.2.3 Personal characteristics

- *The Client Socio-Demographic and Service Receipt Inventory* (CSSRI) (102) records socio-demographic data as well as previous treatment history. This is a comprehensive self-report measure which registers service utilization and cost of treatment for people with mental disorders.

- *Number of previous treatments* is a worksheet asking the patient to fill out information about any previous treatments for OCD, and the characteristics of the previously received treatments.

- Onset and duration of disorder is a specially designed worksheet where the patients record the onset and duration of his/her OCD.

- *Personality Inventory for DSM-5 Brief Form (PID-BF*) is a recently developed personality inventory, which is aimed at assessing dimensions in personality and personality disorders (110).

- *The Warwick-Edinburgh Mental Well-being Scale* (WEMWBS) (104) will be administered to assess mental well-being. This scale has 14 items covering different aspects of mental health related to mental well-being. The WEMWBS has shown good psychometric properties.

- *The Work and Social Adjustment Scale* (W & SAS) (103) is a short questionnaire measuring work status and social adjustment. The scale has good psychometric properties.

- *The Highly Sensitive Person Scale* (HSPS) (80) is a self-report questionnaire which measures aspects of sensitivity towards internal and external stimuli. The psychometric properties of the scale have been evaluated, demonstrating the scale's reliability and validity.

- *The Dispositional Resilience Scale – Revised* (DRS-15-R) (79) is a 15-item self-report instrument measuring resilience to challenges and life-stress. The Norwegian version of the instrument has good psychometric properties (111).

- *The Brief Illness Perception Questionnaire* (BIPQ) (78) will be administered to record illness perception. Although originally developed for measuring perception of somatic illness, the BIPQ has also been adapted to measure perceptions of mental health. The scale has sound psychometric properties.

- *Behavior Rating Inventory of Executive Function – Adult Version (BRIEF-A)* (82) consists of a self-report questionnaire filled out by the patient, and an informant questionnaire filled out by someone in the immediate family of the patient, each consisting of 75 items. It measures several executive functions: inhibition, ability to shift attention, emotional control, self-monitoring, ability to initiate, working memory capacity, ability to plan/organize, task monitoring and organization of materials.

### 7.3 Behavioral test

During each exposure, the patient will be encouraged to approach the anxiety-triggers and taught how to do this without hesitation or "holding back", and rather "lean into the anxiety" (LET-technique). A standardized procedure has been developed including self-report measures of "holding back". The first exposure will be video-taped, as well as the final in which the patient will decide upon the most challenging trigger to be approached and scored by independent raters.

During the exposures the patients will rate how much they are "holding" back from the exposures, using a LET scale (0-100 where 0 indicate no "holding back").

### 7.4 Neuroimaging

Patients at the Bergen site of the study will be asked to undergo brain scanning using structural and functional magnetic resonance imaging at Haukeland University Hospital on the day before treatment, one week after the initial scan and at 3-month follow-up. The scanning procedures will be similar as those of an already approved protocol for non-relapsed OCD patients (REK reference 2015/936/REK Sør-Øst C).

## 8. Procedural consistency

The OCD-team leader at each site will serve as a contact person for the administrative leader of the project to ensure that all practical issues are taken care of.

### 8.1 Project organization

Project organization has been established.

### 8.2 Recruitment and attrition

Standardized study description forms and screening instruments will be used. Refusal rates, attrition rates and reasons will be monitored and compared quarterly.

Before the informed consent is signed, all eligible patients will be presented both with written information as well as watch a video introducing the study. They will be informed that final inclusion is decided upon by the national team of assessors (see below).

After informed consent has been provided and clinical information has been gathered and reported to the investigators using a standardized form, this information will be provided to a national team of assessors who meet weekly. They will assess if the person meets formal inclusion/exclusion criteria in terms of diagnostic criteria, prior Y-BOCS scores and the other inclusion/exclusion criteria listed. Questionable cases will be evaluated in greater detail by two senior investigators, who after reaching consensus will give the final decision of inclusion or exclusion, similar to what has been done in other large-scale studies (112, 113).

### 8.3 Self-report measures

The self-report measures will either be administered by paper and pencil or using online questionnaires in accordance with the official Norwegian regulations. All self-report measures will be administrated by a specially designated team who will monitor responses on a daily basis.

### 8.4 Therapist conducted screening and assessment

All Y-BOCS and SCID interviews for DSM-5 will be conducted by specially trained and independent assessors, blinded for the study-medication.

The following procedures for training of assessors will be employed:

1. The trainees receive theoretical lessons in how to use the scales.
2. The trainees receive practical instructions and video-demonstrations that illustrate different scores on the different items on the scales.
3. Each trainee receives three videotaped interviews which they rate. In order to proceed, the candidate has to demonstrate a minimum of 80 % ”accuracy” (compared to an expert) on 2/3 of the interviews. On SCID it is suggested to employ a kappa-value of 0.80 and on YBOCS a maximum difference of ± 2 points.
4. When step #3 is completed, the trainee has to perform three live interviews with OCD-patients. These will be video-taped and rated by a blinded expert. The requirements are the same as on Step #3.
5. When Step #4 is completed, the assessor can start doing study-interviews.
6. All interviews are taped. Adherence checks will be performed on the 20 first interviews, and afterwards for 20% of the taped interviews. The checks will be conducted monthly and feedback will be given to the assessors. Significant disparities will be corrected through additional interviewer training and adherence monitoring.

Clinical interviews as well as CGI and GAF are administered by a specially trained psychiatrist or psychologist from the local OCD-teams.

### 8.5 Therapist competence and compliance

All the 4-day treatment groups are led by two experts on the 4-day format together with OCD-therapists who must have participated in the extensive national training program (24) or document equivalent level of training.

In each treatment group the ratio between patients and OCD-therapists will be 1:1 and two experts on the 4-day concentrated format (cET) will participate in each group, which implies direct observation of the therapists.

In order to qualify as a cET-expert, therapists must in addition to being an OCD-therapist, have participated and received supervision in a minimum of two cET groups and demonstrate competency in the exposure procedure in accordance with the OCD CORE competencies (Steketee, Foa and Kozak, 2010) relevant for the 4-day format evaluated independently by two cET experts. Specific competencies required for the 4-day format will be recorded and evaluated separately. Evaluation of competency of the 4-days experts will be repeated every 6 months by the aims of video-taped role-played scenarios as well as multiple choice tests covering typical scenarios which will be rated independently by two cET-experts.

During the study, all therapists participating will be evaluated both for competency and adherence independently by the two cET experts who participate in the group each day. Relevant items in the OCD CORE competencies form will be employed, and the evaluation will be done at the end of each of the two days with exposure. Feedback to the therapists will be provided each day, and if the competence of a given therapist is not acceptable, the therapist will be required to act as an observer the remaining time of the group.

### 8.6 Intervention: The concentrated ERP-treatment combined with DCS-administration

All included patients receive the same concentrated ERP (cET) treatment in combination with DCS or placebo. Therapists and participants are blinded to DCS or placebo randomization.

The patients will meet a GCP-trained assistant the second day of the intervention and receive 2 capsules of DCS or placebo. The first is to be taken after 3 hours of the cET. The participant also receives written information about capsule intake ordination and a telephone number if the participant has any questions or to report AE. The second capsule of DCS/ placebo is to be taken 1 h before cET Day 3.

For description of the cET see Havnen et al. (16, 59, 60). The cET format is based on standard ERP-treatment(114) employed in the national implementation project, but with a focus on actively "leaning into the anxiety" consistent with an inhibitory learning understanding of extinction.

The psychoeducation Day 1will be delivered with the aid of a standardized PowerPoint presentation.

The psychoeducation for the families/ relatives (Day 3 of treatment) will be delivered with the aid of a standardized PowerPoint presentation.

# 9. Procedure

### 9.1 Investigator meeting

Before the study is initiated, the investigators, therapists and psychiatric nurses involved in the study will meet and be briefed about the protocol. If impossible to meet face-to-face there will be arranged an online meeting. The meeting will be logged.

### 9.2 Training course in Good Clinical Practice

Before initiating the study, all participant researchers have to complete a certification-course in Good Clinical Practice (GCP).

### 9.3 Recruitment of participants

Since relapsed OCD-patients are not necessarily actively seeking treatment, the trial will be announced in the local papers and on web-sites relevant for OCD-patients. The announcement will inform the patients to contact the OCD-team for further information regarding the referral procedures. Eligible patients will also be referred to the OCD-team by their local psychiatric outpatient clinic. Patients previously treated in an OCD-team will also be informed of the trial. The OCD-teams websites will also feature information about the trial, as well as information on how to get in contact with the investigators.

### 9.4 Inclusion of participants

- All patients referred to an OCD-team, provided that they previously have received OCD-treatment, are screened by a local therapist for inclusion, including Y-BOCS and M.I.N.I interviews.
- If patiens fulfil inclusion criteria, they will watch a video presenting the study and receive written information before informed consent is signed.
- Before the informed consent is signed, the relevant information regarding previous treatment-course is collected (e.g Y-BOCS scores)
- The information is presented to the national team of assessors, and decision regarding inclusion is made.
- Each patient will receive information about the decision, as well as practical details regarding appointments, procedures, treatment and follow-up.

### 9.5 Preparation for treatment

- After inclusion and before treatment, a local OCD therapist conduct an intake interview (anamnestic information and information regarding previous treatment and timeline for relapse), and information regarding the cET is briefly repeated before treatment credibility (CT) is measured.
- The SCID-5 and Y-BOCS are performed within 4 weeks prior to the intervention, by independent and specially trained assessors. For training procedures, see section 8.4.
- The patient fill out relevant self-report questionnaires (see Table 1)

### 9.6 Treatment

- ***Day 1 Psycho-education and treatment preparation (3 h)***

  Participants receive psychoeducation focused on the rationale behind the cET treatment which emphasizes the differences between this approach and the ERP they previously have received.  Relevant exposure tasks will be decided upon. The patients are introduced to the micro-intervention "Leaning into the anxiety" (LET), which is to be practiced throughout the two days of exposure

- ***Day 2 (8 hrs) and 3(8 h + 2 h psychoeducation for family): Therapist assisted Exposure and response prevention combined with DCS/ placebo***

  Patients will engage in individually tailored and therapist assisted ERP, using the micro-intervention LET. Exposures will be conducted in a variety of settings with high ecological relevance, most of them out of the office. DCS on day 2 (first day of exposure) will be taken after 3 hours of the cET. DCS on day 3 will be given approximately 1 hour before the first exposure session of the day.

  On day 3 the patient's family/ significant others are invited to a 2 h psychoeducation.

- ***Day 4: Summarizing and relapse prevention***

Summarizing ("lessons learnt") and planning self-exposures for the next three weeks. Focus will be on teaching the patients how to be "their own therapists. The patients are provided with written information covering procedures and times for upcoming assessments. The patients will, in a group setting, give structured feedback/ evaluation of the treatment at the end, and will prepare exposure tasks to be performed the first three weeks after the treatment.

### 9.7 Homework assignments during the first three weeks post treatment

Participants will log their compliance to the home-work on a daily basis and return the logs to the clinic weekly online or by mail in pre-paid envelopes with return address.

### 9.8 Follow-up visit to the clinic

There will be scheduled an individual follow-up meeting at the clinic three months after the 4-day treatment is completed.

### 9.9 Post-treatment assessments

***After one week (T1)***

**Y-BOCS interview and self-report questionnaires** (see table 1).

***After 3 and 12 months and 5 years.***

SCID-I (at 3 months only OCD module) and Y-BOCS interviews by specially trained assessors as well as a number of self-report instruments (see table 1).

## 10. DCS/ placebo side effects

The DCS will be administrated in two single dosages, and the patients will be randomized to receive either 100 mg or 250 mg. Both dosages are smaller than standard dosage when the drug is administrated for its original purpose.

Previous trials with DCS with doses of 25-250 mg have reported little or no side-effects of DCS compared to placebo (11, 117-119). One potential danger could be that DCS would produce

reversed effects, i.e. DCS could enhance fear learning instead of fear extinction. One study reported facilitation of reconsolidation of fear if DCS was given prior to a single recall trial, but after using several recall trials, it facilitated extinction (120). No other published reports have reported any reversed effects of DCS.

In order to control for possible interactions with antidepressants (121) patients will be stratified in accordance to whether they receive antidepressants or not.

The DCS will be taken at the clinic, administered by a designated person, and the patients will be together with the therapist for at least five hours after intake. This ensures both compliance as well as the possibility to record side effects. Patients will be in contact with the clinic weekly for the first three weeks after the DCS/placebo intervention, which further ensures recording of side effects.

## 11. Measures to minimize bias

The following procedures have been taken to avoid bias:

- This is a double-blind study and the study capsules will be of identical appearance in order to protect the blinding for both participants, therapists, assessors and investigators. As there are no obvious side effects associated with the drug, the placebo capsules will be passive.

- Post-treatment assessments of primary outcomes are conducted by specially trained assessors who are blinded to the treatment conditions and not involved in the treatment (see section 8.4).

### 11.1 Packaging, Labeling, Storage and Handling of Investigational Medicinal Products

| | ERP | DCS 250 mg | DCS 100 mg | Placebo |
|---|---|---|---|---|
| Dosage form | Concentrated, therapist assisted | Capsule DCS 250 mg | Capsule DCS 100 mg | Placebo capsule |
| Unit dosage | 4 days | 250 mg a day, two consecutive days | 100 mg a day, two consecutive days | 250 mg a day, two consecutive days |

### 11.2 Treatment Assignment/ Randomization

Patients are upon inclusion randomly assigned to DCS or placebo using a 2:2:1 ratio of 250 mg DCS: 100 mg DCS: placebo. In order to control for potential interaction effects with antidepressants (121), patients will be stratified on current anti-depressive medication or not. Within each subgroup patients will be randomized in blocks of 5 into ERP+DCS 100 mg, ERP+DCS 250 mg, or ERP + Placebo in the proportions 2:2:1.

A research unit at Karolinska Institute, Sweden, which is not involved in the study, will be responsible for the randomization. Randomization lists will be sent directly to Kragerø pharmacy that will be responsible for providing the medication for patients enrolled.

### 11.3 Blinding and code breaking

Sealed envelopes with the DCS/Placebo treatments will be sent from the Kragerø Tablet Production and given to the medical competent. The envelopes will be kept in a secured place and accessible to any person authorized to un-blind. A decoding list containing the treatment codes will be kept.
The code for any study participant should only be broken by the medical competent or authorized person if it is absolutely necessary to ascertain the type of treatment given. The circumstances under which the code may be broken are life-threatening emergencies, for which the choice of therapy may depend on the treatment received by the patient. If so, the medical competent must write his/ her name, signature, date, the number of the participant concerned and reason for breaking the code on the code envelope. All information concerning adverse drug reactions, drug interactions are given in the SPC [66]. The blinding will be broken after the 12 months follow-up. At the 5-year follow-up the assessors will be blinded for the randomization.

### 11.4 Concomitant and prior medication

The patients are not allowed to use anxiety reducing medication (barbiturates, benzodiazepines, alcohol) in the treatment period (during the two active exposure days). There are no restrictions on the use of dietary supplements or herbal medicines.

### 11.5 Compliance to drug treatment

DCS/ placebo will be administered by designated personnel in order to ensure compliance, and the drug will be taken in front of the therapist.

### 11.6 Product Accountability

Both DCS and placebo will be supplied from Kragerø Tablet Production AS, Storgata 16, 3770 Kragerø, Norway. Tel: +47-35 99 35 50. Treatment management will be under responsibility of the investigator.
The therapeutic DCS units and placebo will be sent to the sites and will be stored in a secure area with restricted access. The expiry date will appear on each box and each label. Placebos are labeled same expiry date as the DCS. Name and phone number of the principal investigator will also appear on the label.

The investigators will acknowledge the receipt of therapeutic units by signing the shipping form. The DCS treatments will be dispensed by the investigators in accordance with the study plan and following dispensing methods described below. Drug accountability will be the responsibility of the investigator. Remaining capsules will be collected by monitor and sent Kragerø Tablet Production AS for destruction.

### 11.7 Continuation of cET-Treatment in discontinuation of DCS-treatment

If a participant decides not to take the DCS/placebo treatment Day 2, he/she has still the opportunity to continue the concentrated ERP treatment, but the patient will be excluded from the study. The whole trial will end when the last subject has completed the 12-month follow-up telephone assessment (separate papers describing the short- and long-term effects of DCS will be written). A 5 year follow-up telephone assessment will be conducted and data will be collected and analyzed seperately.

### 11.8 Unscheduled visits to the clinic

All patients included will be receiving what is part of standard care in the OCD-team. If the OCD should be worsened, or if any other psychiatric problems should arise or be worsened (e.g.

depression) the patients will have the option to contact the OCD team, and the patients will receive the necessary care. Such patients will not be removed from the protocol, but be thoroughly described in the published article of the outcome study.

### 11.9 Discontinuation of the study

The study will be stopped if documentation clearly shows serious side effects related to DCS in two or more patients.

### 11.10 Clinical Safety Assessments

Safety assessments will be performed face-to face at the two days of DCS/ placebo treatment as well as at three using the adverse event questionnaire, as indicated in "Investigational schedule". The same procedure will be done using telephone assessment at 12 months, and 5-year follow-up.

### 11.11 Laboratory Assessments

Patients will be invited to participate in another study involving genotyping samples of blood and saliva, but participation in the present study is not contingent upon participation in the genotyping study.

## 12. Proceedings for Adverse Events

### 12.1 Definition of Adverse Events

An Adverse Event (AE) is any unwanted medical occurrence in a subject administered Investigational Medicinal Products (1) and which does not necessarily have a causal relationship with this product. An AE can be any unfavorable and unintended sign, abnormal laboratory finding, symptom or disease temporally associated with the use of IMP, whether or not related to the product. Known side effects of DCS include mild weariness, drowsiness, headaches and tremor. As DCS has been routinely given in far larger doses in studies of tuberculosis, and we only provide small doses for two consecutive days the known side effects recorded in the SPC will not be reported as AEs.

### 12.2 Definition of Adverse Reactions

Each AE is to be classified by the investigator as related or not related to the IMP. An Adverse Reaction (AR) is a noxious and unintended medical ***response*** to a medical product related to any dose. For an AE to be an AR the suspected association between the product and the unwanted medical condition should be at least a reasonable ***possibility***.

### 12.3 Definition of Serious Adverse Events

Each AE is to be classified by the investigator as serious or non-serious. Seriousness is not defined by a medical term; it is a result or an outcome. An AE is defined as a Serious Adverse Event (SAE) if it:

- results in death
- is life-threatening
- Requires inpatient hospitalization or prolongation of existing hospitalization
- results in persistent or significant disability/incapacity
- results in a congenital anomaly/birth defect

### 12.4 Definition of Suspected Unexpected Serious Adverse Reactions

Each SAE that is at least possibly related to IMP is to be classified by the investigator as expected or unexpected. A SAE that is at least possibly related to IMP, and ***unexpected***, is defined as a Suspected Unexpected Serious Adverse Reaction (SUSAR). It is expected if it is already known from earlier trials or is mentioned in relevant documents. The reference security information of the IMP can be found in the approved SPC (122).

## 12.5 Assessment of Adverse Events

AE is assessed at each clinician visit (including 3 months follow up), and via telephone assessments (12 months and 5 years) and in the treatment platform. If any AE is recorded in the treatment platform, the therapist will print out the information and give it to the investigator.

### 12.5.1 *Assessment of Intensity*

Each AE is to be classified by the investigator as mild, moderate or severe.
**Mild:** Acceptable. The subject is awareness of symptoms or signs, but they are easy tolerated.
**Moderate:** Disturbing. The AE is discomfort enough to interfere with usual daily activity.
**Severe:** Unacceptable. The subject is incapacity to work or to do usual daily activities.

### 12.5.2 *Assessment of Causality*

**Unlikely:** The event is most likely related to an etiology other than the IMP.
**Possible:** A causal relationship is conceivable and cannot be dismissed.
**Probably:** Good reason and sufficient documentation to assume a causal relationship.

## 12.6 Methods for Eliciting Adverse Events

Safety measurements performed for each clinician visit  and telephone assessments (3 months, 12 months and 5 years) (as indicated in "Investigational schedule).

## 12.7 Reporting of Adverse Events

### 12.7.1 *Reporting of Adverse Events*

All AEs will be recorded on a separate AE form in the CRF.

### 12.7.2 *Reporting of Serious Adverse Events*

SAEs will be reported by the investigator and sent the principle investigator immediately. Follow-up information describing the outcome of the SAE and action taken will be reported as soon as it is available. The original SAE form must be filed with the CRF.

### 12.7.3 *Reporting of Suspected Unexpected Serious Adverse reactions*

The sponsor must report all SUSARs that resulted in death or was life threatening to The Norwegian Medicines Agency; NOMA within 7 days. Other SUSAR should be reported within 15 days. In addition will the sponsor report all SUSAR to all Principal Investigators involved in trials with the IMP.

## 12.8 Follow-up of Adverse Events

The investigator must ensure that follow-up of the participant is appropriate to the nature of the event, and that it continues until resolution. Any change in terms of diagnosis, intensity, seriousness, measures taken, causality or outcome regarding adverse events must be written up in an Adverse Events evaluation form.

# 13. Statistics and Data Management

## 13.1 Statistical Analysis

Statistical analysis will be performed by the investigator and specially designated collaborators and quality controlled and cross-checked by independent party

The primary objective is to demonstrate superiority of concentrated ERP + DCS vs. concentrated ERP + placebo at significance level of < 0.05 (5%). Data will basically be analyzed using intention to treat methodology with a mixed models design, accounting for missing data, with relevant covariates for a given analysis. Data will primarily be analyzed employing multivariate methods (ANOVAs/MANOVAs) with repeated measurement.

Covariates are described in the moderator section (Chapter 6.5). Missing data will be replaced (multiple imputation methods). There will be calculations of effect sizes (Cohen's effect size) for all primary and secondary endpoints. Other relevant multivariate statistical analyses will be performed if relevant to elucidate results of importance to the objectives of the study. Safety data (e.g. side effects) will be collected and summarized in the study results.

***Handling of missing data and drop-outs.***

Since the treatment is concentrated over a short number of days, the drop-out rates are, based on our experiences expected to be very low (so far, no patients have dropped out from the intensive treatment). Missing data will be handled by standard procedures.

## 13.2 Determination of Sample Size

A meta-analysis by Norberg, Krystal & Tolin [69] found a medium effect size for ERP + DCS for heterogeneous samples of OCD-patients. However, in the current study, only patients who have relapsed after an initially successful ERP-treatment will be included. This is a sample which supposedly increases the possibility to detect DCS-effects and we assume that the effect size would be larger than that found in previous studies. In order to have 80% power to detect a moderate effect size (d = 0.50) at an alpha-level of 0.05 a total of 160 patients will be included (64 in each of the DCS-conditions and 32 in the Placebo group). Patients lost or withdrawing from the study will be treated as drop-outs and replaced. We estimate an increased sample size of 10 patients, in total, to compensate for possible drop-outs.

# 14. Quality control and Quality Assurance

The investigator will allow the monitors, the persons responsible for the audit, the representatives of the ethics committee and of competent authorities to have direct access to source data and documents.

## 14.1 Source Data

Patient source data file will be established before the study starts. Patient data are stored in the CRF and also as computerized information at the Helse Bergen Research Server.

## 14.2 Monitoring

To ensure compliance with the protocol, monitoring visits by Kristin Odfjell will be scheduled to take place early in the study, during the study at appropriate intervals and after the last subject has completed the study. These visits are for the purpose of confirming that investigator sponsored studies are being conducted in accordance with the ethical principles that have their origins in the Declaration of Helsinki and that that are consistent with Good Clinical Practice and

the applicable regulatory requirements.

### 14.3 Protocol Amendment

A written description and log of change(s) of the protocol will be provided if applicable.

### 14.4 Inspection

The investigator will allow representatives of the competent authorities to conduct an inspection. The persons responsible for the inspection can:

- Inspect the site, facilities and study medication used in the study.
- Meet all members of the team involved in the study.
- Have direct access to study data and source documents.
- Consult all of the documents relevant to the study.

## 15. Ethics

### 15.1 Independent Ethics Committee

The study protocol has been approved of by the Regional Ethical Committee, 2013/195/REK Sør-Øst. Minor changes in the protocol have been reported and approved. Any changes to the protocol that will require approval from the Ethical Committee will be reported, and copies of the applications and decisions will be kept.

### 15.2 Ethical Conduct of the Trial (risks and benefits)

The study will be performed in accordance with the ethical principles stated in the declaration of Helsinki 1964, revised in Seoul 2008. DCS doses in the suggested range have routinely been used in the treatment of tuberculosis for many years. It has not been reported that DCS lessens the effect of ERP/ ERP. If the participants have any inquires about the treatment, they have the possibility to contact a therapist in the treatment platform and expect an answer within 24 hours. Data are gathered in accordance with the Norwegian Personal Data Act and patients give their written consent for data storage. OCD is associated with lowered quality in life, and patients who relapse from a successful ERP-treatment have received little attention in the research literature. These patients have currently few treatment alternatives, and this study might represent invaluable new knowledge that might change the treatment recommendations.

### 15.3 Subject Information and Informed Consent

Therapists in the OCD-teams collect written consent form each participant before participation in the study. Prior to this, the investigator or delegate must inform the patient of the objectives, benefits, risks and requirements imposed by the study, as well as the nature of the study products.

One original of the informed consent will be signed by the participant and collected by the clinician. The participant will receive a copy of the original signed informed consent.

## 16. Data Handling and Record Keeping

### 16.1 Case Report Forms

The investigator designates authorization to other personnel in written form in the CRF (signed by the investigator and date of signature). All changes must be signed by the investigator and

authorized personnel at the clinic. Each CRF should have a copy that is stored at another location. CRF data quality inspector is Kristin Odfjell.

### 16.2 Record Keeping

The investigator is responsible for saving all documents in readable condition for 15 years after the study has been reported to the Norwegian medical products agency. The documents will be accessible for inspection from authorized government agencies during this time.

## 17. Financing and Insurance

The Helse Vest (project no. 911880). The Norwegian Research Council and Norway's Regional Health Authorities (project no. 912013)  fund this study. Patients are insured by the Norwegian patient and medical insurance.

# 18. Publication Policy

Separate plan of publications has been established.

## REFERENCES:


1. Blanco C, Olfson M, Stein DJ, Simpson HB, Gameroff MJ, Narrow WH. Treatment of obsessive-compulsive disorder by U.S. psychiatrists. The Journal of clinical psychiatry. 2006;67(6):946-51.
2. Meyer V. Modification of expectations in cases with obsessional rituals. Behav Res Ther. 1966;4(4):273-80.
3. Abramowitz JSS. Effectiveness of psychological and pharmacological treatments for obsessive-compulsive disorder: a quantitative review. Journal of consulting and clinical psychology. 1997;65(1):44-52.
4. Abramowitz J, Deacon B, J., Whiteside S, P.J. Exposure Therapy for Anxiety: Principles and Practice New York: Guilford Press; 2011.
5. Kvale G, Havik OE, Heiervang ER, Haugland BSM, Tangen T. Hvordan sikre angstpasienter kunnskapsbasert behandling? Oslo: Universitetsforlaget; 2013. 224 p.
6. Perala J, Suvisaari J, Saarni SI, Kuoppasalmi K, Isometsa E, Pirkola S, et al. Lifetime prevalence of psychotic and bipolar I disorders in a general population. Archives of general psychiatry. 2007;64(1):19-28.
7. Knudsen AK, Harvey SB, Mykletun A, Overland S. Common mental disorders and long-term sickness absence in a general working population. The Hordaland Health Study. Acta psychiatrica Scandinavica. 2013;127(4):287-97.
8. Knudsen AK, Overland S, Hotopf M, Mykletun A. Lost working years due to mental disorders: an analysis of the Norwegian disability pension registry. PloS one. 2012;7(8):e42567.
9. Ressler KJ, Rothbaum BO, Tannenbaum L, Anderson P, Graap K, Zimand E, et al. Cognitive enhancers as adjuncts to psychotherapy: use of D-cycloserine in phobic individuals to facilitate extinction of fear. Archives of general psychiatry. 2004;61(11):1136-44.
10. Norberg MM, Krystal JH, Tolin DF. A Meta-Analysis of D-Cycloserine and the Facilitation of Fear Extinction and Exposure Therapy. Biological Psychiatry. 2008;63(12):1118-26.
11. Wilhelm S, Buhlmann U, Tolin DF, Meunier SA, Pearlson GD, Reese HE, et al. Augmentation of behavior therapy with D-cycloserine for obsessive-compulsive disorder. Am J Psychiatry. 2008;165(3):335-41; quiz 409.
12. Storch EA, Murphy TK, Goodman WK, Geffken GR, Lewin AB, Henin A, et al. A Preliminary Study of D-Cycloserine Augmentation of Cognitive-Behavioral Therapy in Pediatric Obsessive-Compulsive Disorder. Biological Psychiatry. 2010;68(11):1073-6.
13. Hofmann SG, Wu JQ, Boettcher H. D-Cycloserine as an augmentation strategy for cognitive behavioral therapy of anxiety disorders. Biol Mood Anxiety Disord. 2013;3(1):11.
14. Mataix-Cols D, Turner C, Monzani B, Isomura K, Murphy C, Krebs G, et al. Cognitive-behavioural therapy with post-session D-cycloserine augmentation for paediatric obsessive-compulsive disorder: pilot randomised controlled trial. Br J Psychiatry. 2014;204(1):77-8.
15. Bontempo A, Panza KE, Bloch MH. D-cycloserine augmentation of behavioral therapy for the treatment of anxiety disorders: a meta-analysis. J Clin Psychiatry. 2012;73(4):533-7.
16. Havnen A, Hansen B, Haug ET, Prescott P, Kvale G. Intensive group treatment for Obsessive-Compulsive Disorder: A pilot study Clinical Neuropsychiatry 2013;10(Supplement).
17. Kozak M, Foa EB. Mastery of Obsessive-Compulsive Disorder. New York: Oxford University Press; 1997.
18. Havnen A, Hansen B, Öst L-G, Kvale G. Intensive and prolonged exposure and response prevention (ERP) for OCD: Individually tailored treatment delivered in a group setting: An effectiveness study. In: R.Dar, M, den Hv, editors. Fourth Meeting of the EABCT SIG on OCD; Assisi2014.

19. Langton JM, Richardson R. D-cycloserine facilitates extinction the first time but not the second time: an examination of the role of NMDA across the course of repeated extinction sessions. Neuropsychopharmacology. 2008;33(13):3096-102.
20. Langton JM, Richardson R. The role of context in the re-extinction of learned fear. Neurobiol Learn Mem. 2009;92(4):496-503.
21. Langton JM, Richardson R. The effect of D-cycloserine on immediate vs. delayed extinction of learned fear. Learning & memory. 2010;17(11):547-51.
22. Langton JM, Richardson R. The temporal specificity of the switch from NMDAr-dependent extinction to NMDAr-independent re-extinction. Behav Brain Res. 2010;208(2):646-9.
23. Ost LG, Havnen A, Hansen B, Kvale G. Cognitive behavioral treatments of obsessive-compulsive disorder. A systematic review and meta-analysis of studies published 1993-2014. Clin Psychol Rev. 2015;40:156-69.
24. Kvale G, Hansen B. Dissemination and intensifying evidence-based treatment for OCD: Norway is in the lead. The Nordic Psychiatrist. 2014:14-5.
25. Ohman A, Mineka S. Fears, phobias, and preparedness: toward an evolved module of fear and fear learning. Psychological review. 2001;108(3):483-522.
26. Phelps EA. Emotion and cognition: insights from studies of the human amygdala. Annual review of psychology. 2006;57:27-53.
27. Sehlmeyer C, Schoning S, Zwitserlood P, Pfleiderer B, Kircher T, Arolt V, et al. Human fear conditioning and extinction in neuroimaging: a systematic review. PloS one. 2009;4(6):e5865.
28. LeDoux JE. Emotion circuits in the brain. Annu Rev Neurosci. 2000;23:155-84.
29. Bolles RC. Species-Specific Defense Reactions and Avoidance Learning. Psychological review. 1970;77(1):32-48.
30. Bolles RC, Fanselow MS. A Perceptual-Defensive-Recuperative Model of Fear and Pain. Behav Brain Sci. 1980;3(2):291-301.
31. Davis M. Role of NMDA receptors and MAP kinase in the amygdala in extinction of fear: clinical implications for exposure therapy. Eur J Neurosci. 2002;16(3):395-8.
32. Davis M. The role of the amygdala in fear and anxiety. Annu Rev Neurosci. 1992;15:353-75.
33. Pavlov IP. Conditioned reflexes. Anrep GV, editor. London: Oxford University Press; 1927.
34. Bouton ME. Context, ambiguity, and unlearning: sources of relapse after behavioral extinction. Biol Psychiatry. 2002;52(10):976-86.
35. Phelps EA, Delgado MR, Nearing KI, LeDoux JE. Extinction learning in humans: role of the amygdala and vmPFC. Neuron. 2004;43:897-905.
36. Bouton ME. Context, ambiguity, and unlearning: sources of relapse after behavioral extinction. Biol Psychiatry. 2002;52:976-86.
37. Rescorla RA. Stimulus generalization: some predictions from a model of Pavlovian conditioning. J Exp Psychol Anim Behav Process. 1976;2(1):88-96.
38. Rescorla RA. Pavlovian conditioning. It's not what you think it is. Am Psychol. 1988;43(3):151-60.
39. Bovet D, Bovet-Nitti F, Oliverio A. Genetic Aspects of Learning and Memory in Mice. Science. 1969;163(3863):139-49.
40. Hodes RL, Cook EW, Lang PJ. Individual Differences in Autonomic Response: Conditioned Association or Conditioned Fear? Psychophysiology. 1985;22(5):545-60.
41. Bouton ME, Mineka S, Barlow DH. A modern learning theory perspective on the etiology of panic disorder. Psychological review. 2001;108(1):4-32.

42. Kvale G, Asbjornsen A, Rosengren B, Lote K, Nordby H, Hammerborg D, et al. Conditioned Nausea and Vomiting in Cancer-Patients - in Search of Mediating Mechanisms. Int J Psychophysiol. 1991;11(1):50-.
43. LaBar KS, Gatenby JC, Gore JC, LeDoux JE, Phelps EA. Human amygdala activation during conditioned fear acquisition and extinction: a mixed-trial fMRI study. Neuron. 1998;20(5):937-45.
44. Hamann S, Canli T. Individual differences in emotion processing. Current opinion in neurobiology. 2004;14(2):233-8.
45. Shackman AJ, Fox AS, Oler JA, Shelton SE, Davidson RJ, Kalin NH. Neural mechanisms underlying heterogeneity in the presentation of anxious temperament. Proceedings of the National Academy of Sciences of the United States of America. 2013;110(15):6145-50.
46. LeDoux J. The Emotional Brain: The Mysterious Underpinnings of Emotional Life Great Britain: Weidenfeld & Nicholson; 1998.
47. Davis M. Facilitation of fear extinction by NMDA agonists. European Neuropsychopharmacology. 2003;13, Supplement 4(0):S172-S3.
48. Davis M, Ressler K, Rothbaum BO, Richardson R. Effects of D-cycloserine on extinction: translation from preclinical to clinical work. Biol Psychiatry. 2006;60(4):369-75.
49. Sotres-Bayon F, Diaz-Mataix L, Bush DE, LeDoux JE. Dissociable roles for the ventromedial prefrontal cortex and amygdala in fear extinction: NR2B contribution. Cerebral cortex. 2009;19(2):474-82.
50. Chang CH, Maren S. Medial prefrontal cortex activation facilitates re-extinction of fear in rats. Learn Mem. 2011;18(4):221-5.
51. Kushner MG, Kim SW, Donahue C, Thuras P, Adson D, Kotlyar M, et al. D-Cycloserine Augmented Exposure Therapy for Obsessive-Compulsive Disorder. Biological Psychiatry. 2007;62(8):835-8.
52. Chasson GS, Buhlmann U, Tolin DF, Rao SR, Reese HE, Rowley T, et al. Need for speed: Evaluating slopes of OCD recovery in behavior therapy enhanced with d-cycloserine. Behaviour Research and Therapy. 2010;48(7):675-9.
53. Siegmund A, Golfels F, Finck C, Halisch A, Räth D, Plag J, et al. d-Cycloserine does not improve but might slightly speed up the outcome of in-vivo exposure therapy in patients with severe agoraphobia and panic disorder in a randomized double blind clinical trial. Journal of Psychiatric Research. 2011;45(8):1042-7.
54. Farrell LJ, Waters AM, Boschen MJ, Hattingh L, McConnell H, Milliner EL, et al. Difficult-to-treat pediatric obsessive-compulsive disorder: feasibility and preliminary results of a randomized pilot trial of D-cycloserine-augmented behavior therapy. Depression and anxiety. 2013;30(8):723-31.
55. Kariuki-Nyuthe C, Gomez-Mancilla B, Stein DJ. Obsessive compulsive disorder and the glutamatergic system. Curr Opin Psychiatry. 2014;27(1):32-7.
56. Rothbaum BO. Critical parameters for D-cycloserine enhancement of cognitive-behaviorial therapy for obsessive-compulsive disorder. Am J Psychiatry. 2008;165(3):293-6.
57. Davis M. NMDA receptors and fear extinction: implications for cognitive behavioral therapy. Dialogues Clin Neurosci. 2011;13(4):463-74.
58. Murray CJ, Lopez AD. Global mortality, disability, and the contribution of risk factors: Global Burden of Disease Study. Lancet. 1997;349(9063):1436-42.
59. Havnen A, Hansen B, Öst L-G, Kvale G. Concentrated ERP delivered in a group setting: An effectiveness study. Journal of Obsessive Compulsive and Related Disorders. 2014;3:319-24.
60. Havnen A, Hansen B, Öst LG, Kvale G. Concentrated ERP delivered in a group setting: Replication and extension. . Manuscript in preperation.
61. Mataix-Cols D, Fernández de la Cruz L, Nordsletten AE, Lenhard F, Isomura K, Simpson HB. Towards an international expert consensus for defining treatment response,

remission, recovery, and relapse in Obsessive-Compulsive Disorder: A Delphi survey. World psychiatry. 2016;in press.
62. Jacobson NS, Truax P. Clinical significance: a statistical approach to defining meaningful change in psychotherapy research. J Consult Clin Psychol. 1991;59(1):12-9.
63. First MB, Spitzer RL, Gibbon M, Williams JBW. The Structured Clinical Interview for DSM IV - Axis 1 disorders (Klinisk version). Danderyd: Pilgrim Press; 1998.
64. van den Heuvel OA, Groenewegen HJ, Barkhof F, Lazeron RH, van Dyck R, Veltman DJ. Frontostriatal system in planning complexity: a parametric functional magnetic resonance version of Tower of London task. Neuroimage. 2003;18(2):367-74.
65. van den Heuvel OA, van der Werf YD, Verhoef KM, de Wit S, Berendse HW, Wolters E, et al. Frontal-striatal abnormalities underlying behaviours in the compulsive-impulsive spectrum. J Neurol Sci. 2010;289(1-2):55-9.
66. van den Heuvel OA, Van Gorsel HC, Veltman DJ, Van Der Werf YD. Impairment of executive performance after transcranial magnetic modulation of the left dorsal frontal-striatal circuit. Hum Brain Mapp. 2013;34(2):347-55.
67. van den Heuvel OA, Veltman DJ, Groenewegen HJ, Witter MP, Merkelbach J, Cath DC, et al. Disorder-specific neuroanatomical correlates of attentional bias in obsessive-compulsive disorder, panic disorder, and hypochondriasis. Arch Gen Psychiatry. 2005;62(8):922-33.
68. van den Heuvel OA, Veltman DJ, Groenewegen HJ, Cath DC, van Balkom AJ, van Hartskamp J, et al. Frontal-striatal dysfunction during planning in obsessive-compulsive disorder. Arch Gen Psychiatry. 2005;62(3):301-9.
69. van den Heuvel OA, Mataix-Cols D, Zwitser G, Cath DC, van der Werf YD, Groenewegen HJ, et al. Common limbic and frontal-striatal disturbances in patients with obsessive compulsive disorder, panic disorder and hypochondriasis. Psychol Med. 2011;41(11):2399-410.
70. Szeszko PR, Christian C, Macmaster F, Lencz T, Mirza Y, Taormina SP, et al. Gray matter structural alterations in psychotropic drug-naive pediatric obsessive-compulsive disorder: an optimized voxel-based morphometry study. Am J Psychiatry. 2008;165(10):1299-307.
71. Menzies L, Chamberlain SR, Laird AR, Thelen SM, Sahakian BJ, Bullmore ET. Integrating evidence from neuroimaging and neuropsychological studies of obsessive-compulsive disorder: The orbitofronto-striatal model revisited. Neuroscience & Biobehavioral Reviews. 2008;32(3):525-49.
72. Delis DC, Kaplan E, Kramer JH. Delis-Kaplan executive function system (D-KEFS): Examiner’s manual. . San Antonia, TX: Psychological Corporation.; 2001.
73. Borkovec TD, Nau SD. Credibility of analogue therapy rationales. J Behav Ther Exp Psychiatry. 1972;3(4):257-60.
74. Ryder AG, Costa PT, Bagby RM. Evaluation of the SCID-II personality disorder traits for DSM-IV: coherence, discrimination, relations with general personality traits, and functional impairment. Journal of personality disorders. 2007;21(6):626-37.
75. First MB, Spitzer RL, Gibbon M, Williams JBW. Structured Clinical Interview for DSM-IV Axis I Disorders-Patient Edition (SCID-I/P, Version 2.0). Biometric Research Departement, New York State Psychiatric Institute: New York. 1995.
76. Munder T, Wilmers F, Leonhart R, Linster HW, Barth J. Working Alliance Inventory-Short Revised (WAI-SR): psychometric properties in outpatients and inpatients. Clin Psychol Psychother. 2010;17(3):231-9.
77. Pallesen S, Bjorvatn B, Nordhus IH, Sivertsen B, Hjornevik M, Morin CM. A new scale for measuring insomnia: the Bergen Insomnia Scale. Perceptual and motor skills. 2008;107(3):691-706.

78. Petrie KJ, Weinman J, Sharpe N, Buckley J. Role of patients' view of their illness in predicting return to work and functioning after myocardial infarction: longitudinal study. BMJ. 1996;312(7040):1191-4.
79. Bartone PT. Test-retest reliability of the dispositional resilience scale-15, a brief hardiness scale. Psychol Rep. 2007;101(3 Pt 1):943-4.
80. Aron EN, Aron A. Sensory-processing sensitivity and its relation to introversion and emotionality. Journal of personality and social psychology. 1997;73(2):345-68.
81. Kessler RC, Adler L, Ames M, Demler O, Faraone S, Hiripi E, et al. The World Health Organization Adult ADHD Self-Report Scale (ASRS): a short screening scale for use in the general population. Psychol Med. 2005;35(2):245-56.
82. Roth RM, Isquith PK, Gioia GA. Behavior Rating Inventory of Executive Function–Adult Version (BRIEF-A). Lutz: Psychological Assessment Resources; 2005.
83. Sheehan DV, Lecrubier Y, Sheehan KH, Amorim P, Janavs J, Weiller E, et al. The Mini-International Neuropsychiatric Interview (MINI): the development and validation of a structured diagnostic psychiatric interview for DSM-IV and ICD-10. Journal of clinical psychiatry. 1998;59:22-33.
84. Sheehan D, Lecrubier Y, Sheehan KH, Janavs J, Weiller E, Keskiner A, et al. The validity of the Mini International Neuropsychiatric Interview (MINI) according to the SCID-P and its reliability. European Psychiatry. 1997;12(5):232-41.
85. Spitzer RL, Williams JB, Gibbon M, First MB. The Structured Clinical Interview for DSM-III-R (SCID). I: History, rationale, and description. Arch Gen Psychiatry. 1992;49(8):624-9.
86. Williams JB, Gibbon M, First MB, Spitzer RL, Davies M, Borus J, et al. The Structured Clinical Interview for DSM-III-R (SCID). II. Multisite test-retest reliability. Archives of general psychiatry. 1992;49(8):630-6.
87. Goodman WK, Price LH, Rasmussen SA, Mazure C, Fleischmann RL, Hill CL, et al. The Yale-Brown Obsessive Compulsive Scale. I. Development, use, and reliability. Arch Gen Psychiatry. 1989;46(11):1006-11.
88. Baer L, Blais MA. Handbook of clinical rating scales and assessment in psychiatry and mental health. New York: Humana Press; 2010. xxi, 320 p. p.
89. American Psychiatric Association. Diagnostic and statistical manual of mental disorders : DSM-IV-TR. 4th ed. Washington, DC: American Psychiatric Association; 2000. xxxvii, 943 p. p.
90. Busner J, Targum SD. The clinical global impressions scale: applying a research tool in clinical practice. Psychiatry. 2007;4(7):28-37.
91. Foa EB, Huppert JD, Leiberg S, Langner R, Kichic R, Hajcak G, et al. The Obsessive-Compulsive Inventory: development and validation of a short version. Psychol Assess. 2002;14(4):485-96.
92. Abramowitz J, Tolin D, Diefenbach G. Measuring change in OCD: Sensitivity of the Obsessive Compulsive Inventory-Revised. Journal of Psychopathology and Behavioral Assessment. 2005;27: 317-24.
93. Abramowitz JS, Deacon BJ, Olatunji BO, Wheaton MG, Berman NC, Losardo D, et al. Assessment of obsessive-compulsive symptom dimensions: Development and evaluation of the Dimensional Obsessive-Compulsive Scale. Psychological Assessment. 2010;22(1):180-98.
94. Delgadillo J, Payne S, Gilbody S, Godfrey C, Gore S, Jessop D, et al. Brief case finding tools for anxiety disorders: validation of GAD-7 and GAD-2 in addictions treatment. Drug and alcohol dependence. 2012;125(1-2):37-42.
95. Spitzer RL, Kroenke K, Williams JB, Lowe B. A brief measure for assessing generalized anxiety disorder: the GAD-7. Archives of internal medicine. 2006;166(10):1092-7.
96. Swinson RP. The GAD-7 scale was accurate for diagnosing generalised anxiety disorder. Evidence-based medicine. 2006;11(6):184.

97. Andrusyna TP, Tang TZ, DeRubeis RJ, Luborsky L. The factor structure of the working alliance inventory in cognitive-behavioral therapy. J Psychother Pract Res. 2001;10(3):173-8.
98. Cameron IM, Reid IC, Lawton K. PHQ-9: sensitivity to change over time. The British journal of general practice : the journal of the Royal College of General Practitioners. 2010;60(576):535-6.
99. Cannon DS, Tiffany ST, Coon H, Scholand MB, McMahon WM, Leppert MF. The PHQ-9 as a brief assessment of lifetime major depression. Psychol Assess. 2007;19(2):247-51.
100. Hansson M, Chotai J, Nordstom A, Bodlund O. Comparison of two self-rating scales to detect depression: HADS and PHQ-9. The British journal of general practice : the journal of the Royal College of General Practitioners. 2009;59(566):e283-8.
101. Inoue T, Tanaka T, Nakagawa S, Nakato Y, Kameyama R, Boku S, et al. Utility and limitations of PHQ-9 in a clinic specializing in psychiatric care. BMC psychiatry. 2012;12:73.
102. Chisholm D, M. R. J. , Knapp, al. e. Client Socio-Demographic and Service Receipt Inventory - European Version : development of an instrument for international research. The British Journal of Psychiatry 2000;177:28-33.
103. Mundt JC, Marks IM, Shear MK, Greist JH. The Work and Social Adjustment Scale: a simple measure of impairment in functioning. The British journal of psychiatry : the journal of mental science. 2002;180:461-4.
104. Tennant R, Hiller L, Fishwick R, Platt S, Joseph S, Weich S, et al. The Warwick-Edinburgh Mental Well-being Scale (WEMWBS): development and UK validation. Health and quality of life outcomes. 2007;5:63.
105. Keijsers GP, Schaap CP, Hoogduin C, Hoogsteyns B, de Kemp E. Preliminary results of a new instrument to assess patient motivation for treatment in cognitive-behaviour therapy. Behavioural and Cognitive Psychotherapy. 1999;27(02):165-79.
106. Borkovec TD, Nau SD. Credibility of analouge therapy rationales. J Behav Ther Exp Psychiatry. 1972;3:257-60.
107. Larsen DL, Attkisson CC, Hargreaves WA, Nguyen TD. Assessment of client/patient satisfaction: development of a general scale. Evaluation and program planning. 1979;2(3):197-207.
108. Simpson HB, Maher M, Page JR, Gibbons CJ, Franklin ME, Foa EB. Development of a Patient Adherence Scale for Exposure and Response Prevention Therapy. Behavior therapy. 2010;41(1):30-7.
109. Simpson HB, Maher MJ, Wang Y, Bao Y, Foa EB, Franklin M. Patient adherence predicts outcome from cognitive behavioral therapy in obsessive-compulsive disorder. Journal of Consulting and Clinical Psychology. 2011;79(2):247-52.
110. Krueger RF, Derringer J, Markon KE, Watson D, Skodol AE. Initial Construction of a Maladaptive Personality Trait Model and Inventory for DSM-5. Psychological medicine. 2012;42:1879-90.
111. Hystad SW, Eid J, Johnsen BH, Laberg JC, Bartone TP. Psychometric properties of the revised Norwegian dispositional resilience (hardiness) scale. Scandinavian Journal of Psychology. 2010;51(3):237-45.
112. Freeman J, Garcia A, Benito K, Conelea C, Sapyta J, Khanna M, et al. The pediatric obsessive compulsive disorder treatment study for young children (POTS Jr): developmental considerations in the rationale, design, and methods. Journal of obsessive-compulsive and related disorders. 2012;1:294-300.
113. Freeman JB, Choate-Summers ML, Garcia AM, Moore PS, Sapyta JJ, Khanna MS, et al. The Pediatric Obsessive-Compulsive Disorder Treatment Study II: rationale, design and methods. Child and adolescent psychiatry and mental health. 2009;3:4.

114. Foa EB, Yadin E, Lichner TK. Exposure and Response (Ritual) Prevention for Obsessive-Compulsive Disorder: Therapist Guide Oxford University Press; 2012.
115. Guy W, Bonato RR. CGI: Clinical Global Impressions. Manual for the ECDEU Assessment Battery.2 National Institute of Mental Health; 1970.
116. Havnen A, Hansen B, Kvale G, Haug ET, Prescott P, Frotjold R. Intensive group treatment of Obsessive-Compulsive Disorder: A case series. . in preparation.
117. Kushner MG, Kim SW, Donahue C, Thuras P, Adson D, Kotlyar M, et al. D-cycloserine augmented exposure therapy for obsessive-compulsive disorder. Biol Psychiatry. 2007;62(8):835-8.
118. Otto MW, Tolin DF, Simon NM, Pearlson GD, Basden S, Meunier SA, et al. Efficacy of d-cycloserine for enhancing response to cognitive-behavior therapy for panic disorder. Biol Psychiatry.67(4):365-70.
119. Hofmann SG, Meuret AE, Smits JA, Simon NM, Pollack MH, Eisenmenger K, et al. Augmentation of exposure therapy with D-cycloserine for social anxiety disorder. Archives of general psychiatry. 2006;63(3):298-304.
120. Lee JL, Milton AL, Everitt BJ. Reconsolidation and extinction of conditioned fear: inhibition and potentiation. J Neurosci. 2006;26(39):10051-6.
121. Andersson E, Hedman E, Enander J, Radu Djurfeldt D, Ljotsson B, Cervenka S, et al. d-Cycloserine vs Placebo as Adjunct to Cognitive Behavioral Therapy for Obsessive-Compulsive Disorder and Interaction With Antidepressants: A Randomized Clinical Trial. JAMA Psychiatry. 2015;72(7):659-67.
122. Drugs.com. Cycloserine (SPC) 2012 [cited 2012-03-09]. Available from: http://www.drugs.com/uk/cycloserine-spc-8124.html.

# APPENDIX

## Table 1. Measurements employed in the study

| Description | T0 Pretreatment/ baseline | During treatment | T1 One week post treatment | T2 3 months post treatment | T3 One year post treatment | T4 5 years post treatment |
|---|---|---|---|---|---|---|
| Informed consent | X | | | | | |
| Inclusion/exclusion evaluation | X | | | | | |
| Adverse event | | X | X | X | X | X |
| **Assessor administrated instruments** | | | | | | |
| SCID-I | X | | | X | X | X |
| Y-BOCS interview | X | | X | X | X | X |
| GAF | X | | | | X | X |
| CGI | X | | | | | X |
| M.I.N.I | X | | | X | X | X |
| Anamnestic information and interview regarding latest treatment course. | X | | | | | |
| **Treatment characteristics** | | | | | | |
| TC | X | X | | | | |
| Evaluation of the treatment | | X | | | | |
| WAI | | X | | | | |
| LET | | X | | | | |
| CSQ-8 | | | X | | | |
| PEAS | | X | X[1] | | | |
| Prioritized exposure tasks | | | X[1] | | | |
| Daily recording of exposure tasks | | | X[1] | | | |
| **Self-reports of symptoms and disorders** | | | | | | |
| OCI-R | X | X | X | X | X | X |
| GAD-7 | X | X | X | X | X | X |
| DOCS-SF | X | X | X | X | X | X |
| PHQ-9 | X | X | X | X | X | X |
| BIS | X | | X | X | X | X |
| Record of concomitant drug/medication | | | X | X | X | X |
| Y-BOCS checklist | X | | | | | |
| NML-2 | X | | | | | |
| **Personal characteristics** | | | | | | |
| CSSRI | X | | | X | X | X |
| Number of previous treatments | X | | | | | |
| PID-BF | X | | | | X | X |
| WEMWBS | X | | | | X | X |
| W & SAS | X | | | | X | X |
| HSPS | X | | | | X | X |
| DRS-15-R | X | | | | X | X |
| BIPQ | X | | | | X | X |
| BRIEF[2] | X | | | | X | X |

[1] Including 3 weeks post intervention
[2] Both the patient and a person that knows them well will be asked to fill out the BRIEF.

**D-Cycloserine, substance information**

Information on ingredients:
**Formula:** $C_3H_6N_2O_2$
**Chemical Name:** 3-Isoxazolidinone, 4-amino-, (R)-
**CAS:** 68-41-7
**RTECS Number:** NY2975000
**Chemical Family:** Isoxazole
**Therapeutic Category:** Antibacterial (tuberculostatic)
**Autoignition Temperature:** n/f
**ATC Code for cycloserine: J04AB01**
**Formula:** $C_3H_6N_2O_2$
**Molecular Weight:** 102.09

Toxicological properties:
**Oral Rat:** LD50: >5 grams/kg
**Oral Mouse:** LD50: 5290 mg/kg
**Other Toxicity Data:**
Oral Guinea Pig: LD50: > 2 grams/kg
Oral Dog: LD50: > 2 grams/kg
**Irritancy Data:** Rabbit/eye: slight
Rabbit/skin: non-irritating
**Listed as a Carcinogen by: NTP:** No **IARC:** No **OSHA:** No
**Other Carcinogenicity Data:** No
**Mutagenicity Data:** The Ames test and unscheduled DNA repair test were negative
**Reproductive and Developmental Effects:** A study in rats at doses up to 100 mg/kg/day showed no adverse reproductive effects

Disposal
Dispose of waste in accordance with all applicable Federal, State and local laws.
