# Supplementary material for: Effect of D-Cycloserine on the Effect of Concentrated Exposure and Response Prevention in Difficult-to-Treat Obsessive-Compulsive Disorder: A Randomized Clinical Trial
Source: JAMA Netw Open. 2020 Aug 13;3(8):e2013249. doi: 10.1001/jamanetworkopen.2020.13249 (PMC7426745; doi:10.1001/jamanetworkopen.2020.13249)
Supplement: Supplement 2. — eFigure 1. Outline of the 4-Day Treatment eFigure 2. Change Trajectory by Condition for the Yale-Brown Obsessive-Compulsive Scale eFigure 3. Change Trajectory by Relapse and Nonresponse for the Yale-Brown Obsessive-Compulsive Scale eTable. Clinical Improvement According to the Jacobson and Truax Criteria at Posttreatment and Follow-up [file jamanetwopen-3-e2013249-s002.pdf]

Supplementary Online Content

Kvale G, Hansen B, Hagen K, et al. Effect of D-cycloserine on the effect of concentrated exposure and response prevention in difficult-to-treat obsessive-compulsive disorder: a randomized clinical trial. *JAMA Netw Open*. 2020;3(8):e2013249. doi:10.1001/jamanetworkopen.2020.13249

eFigure 1. Outline of the 4-Day Treatment

eFigure 2. Change Trajectory by Condition for the Yale-Brown Obsessive-Compulsive Scale

eFigure 3. Change Trajectory by Relapse and Nonresponse for the Yale-Brown Obsessive-Compulsive Scale

eTable. Clinical Improvement According to the Jacobson and Truax Criteria at Posttreatment and Follow-up

This supplementary material has been provided by the authors to give readers additional information about their work.

© 2020 Kvale G et al. *JAMA Network Open.*

eFigure 1. Outline of the 4-Day Treatment

Time	Day 1	Day 2	Day 3	Day 4
0830-0900		Repetition of psychoeducation	**Study medication**	Sharing experiences from previous evening (in group)
0900-0930		Practical introduction of the LET-intervention	Sharing experiences from previous evening (in group)	Psychoeducation: How to make sure the change is integrated into normal living
0930-1000		Therapist-assisted exposure (individually)	Therapist-assisted exposure (individually)	
1000-1030				Summarizing “lessons learnt”
1030-1100				
1100-1130				Planning exposure the coming 3 weeks
1130-1200				
1200-1230	Psychoeducation (in group)	Lunch with sharing of experiences and feedback from therapists (in group)		Lunch with sharing of experiences and feedback from patients
1230-1300		**Study medication**	Lunch with sharing of experiences and feedback (in group)	
1300-1330		Therapist-assisted exposure (individually)	Therapist-assisted exposure (individually)	
1330-1400	Planning of individualized exposure (in group)			
1400-1430				
1430-1500				
1500-1530				
1530-1600			Sharing of experiences/feedback in group	
1600-1630		Sharing of experiences/feedback (in group)	Psychoeducation for family/relatives (in group)	
1630-1800		Planned self-exposure		
1800-2100			Planned self-exposure	
2100		Text message to therapist	Text message to therapist	

© 2020 Kvale G et al. *JAMA Network Open.*

eFigure 2. Change Trajectory by Condition for the Yale-Brown Obsessive-Compulsive Scale

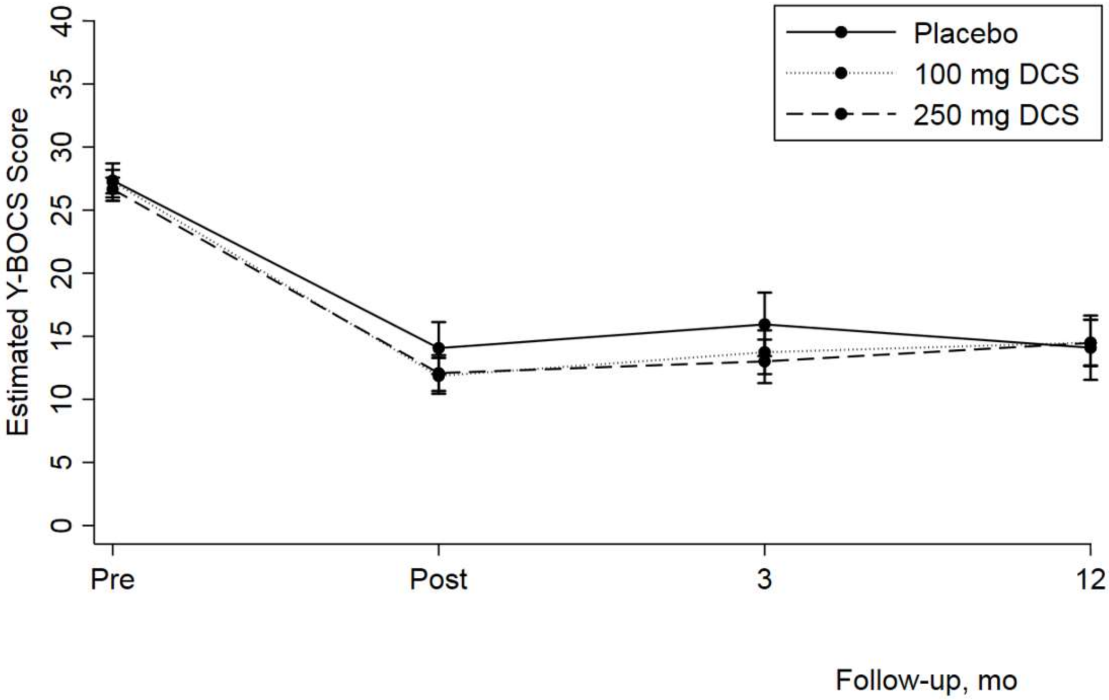

Note. Error bars indicate 95% confidence intervals.

© 2020 Kvale G et al. *JAMA Network Open.*

eFigure 3. Change Trajectory by Relapse and Nonresponse for the Yale-Brown Obsessive-Compulsive Scale

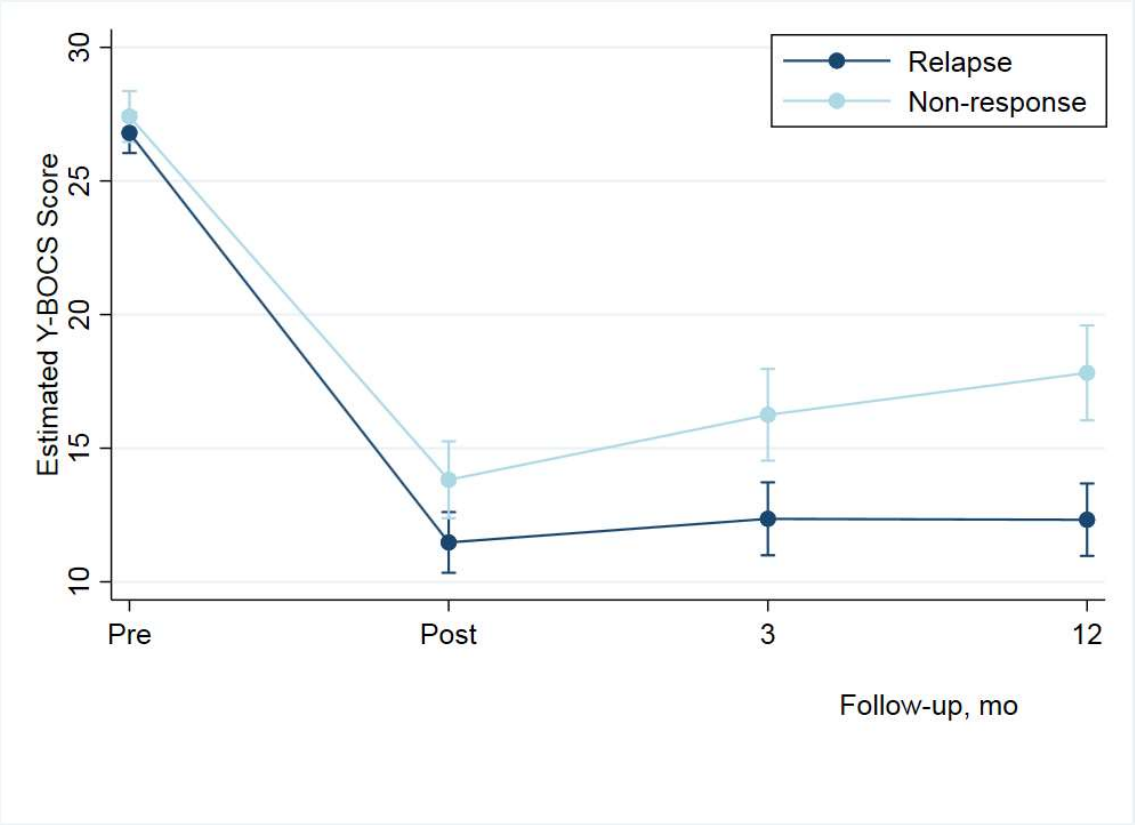

Note. Error bars indicate 95% confidence intervals.

© 2020 Kvale G et al. *JAMA Network Open.*

eTable. Clinical Improvement According to the Jacobson and Truax Criteria at Posttreatment and Follow-up

	**Post-treatment**					**12-month follow-up**			
**Condition**	**CSC**	**RCI**	**No change**	**N**		**CSC**	**RCI**	**No change**	**N**
DCS 250mg	32 (49.2)	30 (46.2)	3 (4.6)	65		25 (41.0)	27 (44.3)	9 (14.7)	61
DCS 100mg	29 (44.6)	35 (53.8)	1 (1.5)	65		23 (40.4)	25 (43.9)	9 (15.8)	57
Placebo	15 (48.4)	13 (41.9)	3 (9.7)	31		10 (35.7)	14 (50.0)	4 (14.3)	28
Total	76 (47.2)	78 (48.4)	7 (4.4)	161		58 (39.7)	66 (45.2)	22 (15.1)	146

Note. DCS = d-cycloserine , CSC = clinically significant change, RCI = reliable change index.

© 2020 Kvale G et al. *JAMA Network Open.*
