# Supplementary material for: Effect of D-Cycloserine on the Effect of Concentrated Exposure and Response Prevention in Difficult-to-Treat Obsessive-Compulsive Disorder: A Randomized Clinical Trial
Source: JAMA Netw Open. 2020 Aug 13;3(8):e2013249. doi: 10.1001/jamanetworkopen.2020.13249 (PMC7426745; doi:10.1001/jamanetworkopen.2020.13249)
Supplement: Supplement 3. — Data Sharing Statement [file jamanetwopen-3-e2013249-s003.pdf]

Data Sharing Statement

Kvale. Effect of D-Cycloserine on the Effect of Concentrated Exposure and Response Prevention in Difficult-to-Treat Obsessive-Compulsive Disorder. *JAMA Netw Open*. Published August 13, 2020.
10.1001/jamanetworkopen.2020.13249

Data
Data available: No

Additional Information
Explanation for why data not available: The GDPR regulations prohibit data sharing
